# Millipede and centipede assemblages on the northern and southern slopes of the lowland Altais, southwestern Siberia, Russia (Diplopoda, Chilopoda)

**DOI:** 10.3897/zookeys.741.21936

**Published:** 2018-03-07

**Authors:** Pavel S. Nefediev, Gyulli Sh. Farzalieva, Ivan H. Tuf, Khozhiakbar Kh. Nedoev, Saparmurad T. Niyazov

**Affiliations:** 1 Department of Ecology, Biochemistry and Biotechnology, Altai State University, Lenina avenue 61, Barnaul,656049, Russia; 2 Biological Institute, Tomsk State University, Lenina avenue 36, Tomsk, 634050, Russia; 3 Department of Invertebrate Zoology and Aquatic Ecology, Perm State University, Bukireva street 15, Perm, 614600, Russia; 4 Department of Ecology and Environmental Sciences, Faculty of Science, Palacký University, Šlechtitelů 27, Olomouc, 77900, Czech Republic

**Keywords:** Altai, millipedes, centipedes, distribution, ecology, lowland, new records, Siberia

## Abstract

The total species richness in the myriapod assemblages of the lowland Altais near Charyshskoe Village, Altai Province, southwestern Siberia, Russia is estimated to be at least 19 species from ten genera, eight families, five orders, and two classes. The following species are new to SW Siberia: Lithobius (Ezembius) ostiacorum Stuxberg, 1876, *L. vagabundus* Stuxberg, 1876, and L. (Monotarsobius) nordenskioeldii Stuxberg, 1876, while L. (E.) proximus Sseliwanoff, 1880 and L. (M.) insolens Dányi & Tuf, 2012 are recorded for the first time from the Altai Province of Russia. A species of *Strigamia* which is morphologically similar to Strigamia cf. transsilvanica (Verhoeff, 1928) has been found in the study area but its true specific identity is yet to be determined. The seasonal dynamics of myriapod assemblages in terms of the species diversity, density, sex-age structure, and vertical distribution along the soil profile have been studied with regard to the different slope exposures.

## Introduction

Despite the recent increased interest in the myriapod fauna of southwestern Siberia (Mikhaljova et al. 2007, 2008, 2014, 2015, Mikhaljova 2009, 2013, 2016, 2017, Nefediev et al. 2013, 2014a, b, c, 2016a, b, c, 2017a, b, Nefedieva et al. 2014, 2015, Nefediev 2016), the biodiversity and ecological characteristics of myriapods in the study area of the lowland Altais, a transition zone from the plains of the southwestern Siberia to the mountains of southern Siberia have not been studied to date.

## Materials and methods

**Figure 1.**
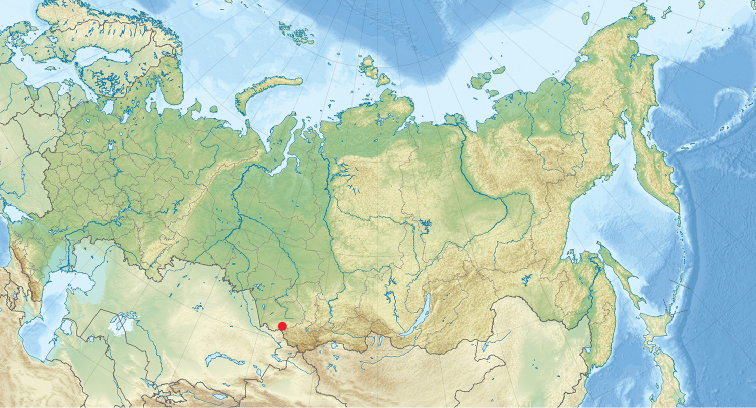
Map of study locality (shown by the red spot).

**Figures 2–5.**
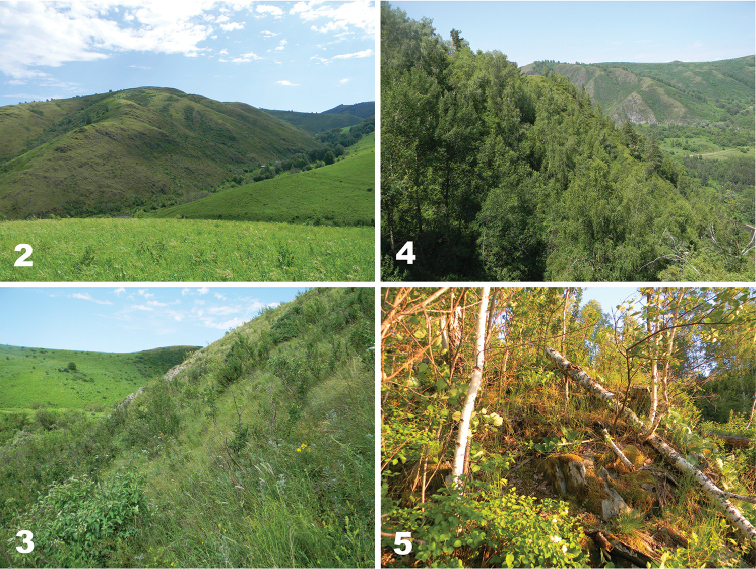
Two types of study habitats. **2–3** rocky xeromorphic steppe with bushes on the southern slope **4–5** rocky forested sites on the northern slope (**2–3** taken in mid-July 2017, **5** taken at the end of May 2016; all by P.N.).

The present study is based on fresh samples collected in the lowlands of the Charysh District, Altai Province, SW Siberia. The area has a continental climate, with cold and snowy winters, and hot and dry summers: mid-temperature in January is –17°C and in July +18.5°C; annual amount of precipitation is about 600 mm. Material from the environs of the Altai State University Student Field Station, titled “Goluboi Utios” (= “Blue Rock” in English), situated ca. 4.5 km SE of Charyshskoye Village (Figure 1) was collected. The vast majority of study material was obtained from two types of habitat. Two sites were sampled in each habitat:

(1) rocky xeromorphic steppe with bushes of Siberian peashrub (*Caragana arborescens*), Tartarian honeysuckle (*Lonicera tatarica*) and germander meadowsweet (*Spiraea chamaedryfolia*) located on the southern slope (Figures 2, 3): site 1 on S slope (51°21'20.3"N, 83°37'36.5"E, 480 m a.s.l.) and site 2 on S slope (51°21'14.5"N, 83°38'03.8"E, 530 m a.s.l.);

(2) rocky forested sites with silver birch (*Betula pendula*), Scots pine (*Pinus sylvestris*), germander meadowsweet (*S. chamaedryfolia*) and Korean elephant-ear, or badan (*Bergenia crassifolia*) on the northern slope (Figures 4, 5): site 1 on N slope (51°21'44.3"N, 83°37'42.6"E, 620 m a.s.l.) and site 2 on N slope (51°21'38.0"N, 83°38'02.7"E, 630 m a.s.l.).

The material was collected using the standard soil fauna sampling techniques practiced in Russia (Ghilarov 1987) by taking 5 soil samples per study site, hand-sorting each 10 cm layer down to 30 cm until fauna penetration, with the sample area totaling ¼ m^2^. Soil samples were taken three times during summer 2016, starting at the beginning of summer (31 May–2 June), through mid-summer (12–13 July) to late summer (22–23 August). Also we collected additional faunistic material in nearby localities by hand sampling in the summers of 2015–2017. The total number of studied millipedes and centipedes was 684 and 666 specimens, respectively.

The distribution of recorded species in soil samples was analyzed using CANOCO for Windows 4.5 (ter Braak and Šmilauer 1998). Following lengths of gradient in species data we selected Redundancy analyses (RDA) using environmental variables, i.e. exposure (south/north), month, depth of soil sample and sample ID. The significance of models was evaluated using Monte Carlo tests with 499 permutations. For the evaluation of significance and effect of tested environmental variables forward selection was applied. The effect of selected significant environmental variables (month, depth) for predicting the distribution of individual species was tested using Generalized linear models (GLM) with evaluation of AIC.

The material treated here was collected by A.M. Alenov (A.A.), E.V. Andreeva (E.A.), Kh.Kh. Nedoev (Kh.N.), P.S. Nefediev (P.N.), S.T. Niyazov (S.N.), V.Yu. Slatina (V.S.), and T.A. Zakirov (T.Z.) (all from Barnaul). These samples have been deposited mainly in the collection of the Altai State University, Barnaul, Russia, Barnaul, Russia (ASU), and shared also with the collection of the Perm State University, Perm, Russia (PSU) and Zoological Museum of the Moscow Lomonosov State University, Moscow, Russia (ZMMU), as indicated in the text. The species names documented in the literature references include those from southwestern Siberia (Asian Russia) only.

## Taxonomic part

### Class Diplopoda de Blainville in Gervais, 1844

#### Order Julida Brandt, 1833

##### Family Julidae Leach, 1814

###### Genus *Leptoiulus* Verhoeff, 1894

####### Leptoiulus tigirek

Taxon classificationAnimaliaJulidaJulidae

Mikhaljova, Nefediev, Nefedieva & Dyachkov, 2015

Figure 6

Julidae gen. sp. – Dyachkov 2014: 41.
undescribed species of Julidae – Nefediev et al. 2014a: 63.
Leptoiulus tigirek Mikhaljova, Nefediev, Nefedieva & Dyachkov 2015: 268, 269–273: figs.
Leptoiulus tigirek – Nefediev 2016: 30; Mikhaljova 2017: 77, 78: figs, insets 733–740, 789, 790, 90: map; Nefediev et al. 2017c: 13.

######## Material examined

(all from Russia, southwestern Siberia, Altai Province, Charysh District, ca. 4.5 km SE of Charyshskoye Village). 1 ♀ (ASU), site 2 on N slope, soil sample 1 (10–20 cm deep), 2.06.2016; 1 ♀ (ASU), site 2 on N slope, soil sample 3 (litter), 2.06.2016, all leg. P.N., Kh.N., S.N., V.S.; 1 ♀ (ASU), *Betula pendula* and *Populus tremula* stand on N slope, 51°21'33.8"N, 83°37'23.2"E, 518 m a.s.l., pitfall traps, 12–14.07.2016, leg. P.N.; 1 ♂ (ASU), site 2 on N slope, soil sample 3 (0–10 cm deep), 13.07.2016; 1 ♂ (ZMUM), 1 ♂, 1 juv. (ASU), site 2 on N slope, hand sampling, 13.07.2016, all leg. Kh.N., S.N., V.S.; 1 ♀ (ASU), site 2 on N slope, hand sampling, 23.08.2016, all leg. P.N., Kh.N., S.N., V.S.; 1 ♀ (ZMUM), 5 ♀♀, 1 juv. (ASU), site 2 on N slope, hand sampling, 23.06.2017, leg. P.N., Kh.N., A.A., E.A.

**Figure 6–7.**
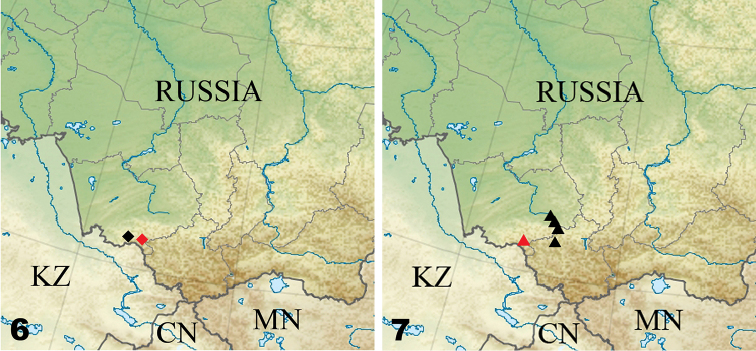
Range limits of some millipede species in the study area. **6** Distribution of *Leptoiulus tigirek* (diamond) **7** Distribution of *Sibiriulus latisupremus* (triangle). The new localities are shown in red.

######## Distribution.

Being an Altai endemic, the species has been recorded only in the Altai Province in southwestern Siberia (Mikhaljova et al. 2015; Nefediev 2016).

######## Remarks.

The julid *L. tigirek* has been collected outside its *terra typica* for the first time. The above records on the northern slope show the species preference for more humid habitats.

###### Genus *Megaphyllum* Verhoeff, 1894

####### Megaphyllum sjaelandicum

Taxon classificationAnimaliaJulidaJulidae

(Meinert, 1868)

Megaphyllum sjaelandicum (Meinert, 1868) – Mikhaljova et al. 2007: 62, fig; Nefediev and Nefedieva 2007b: 162; 2008b: 62; Babenko et al. 2009: 183; Mikhaljova 2013: 9; 2016: 7; 2017: 97, 98: figs, 56: map; Nefediev et al. 2014a: 63; 2017c: 13.

######## Material examined

(all from Russia, southwestern Siberia, Altai Province, Charysh District, ca. 4.5 km SE of Charyshskoye Village). 16 juv. (ASU), site 1 on S slope, 13.07.2015; 1 ♂, 1 ♀, 1 juv. (ZMUM), *Betula pendula* and *Populus tremula* stand on N slope, 51°21'33.8"N, 83°37'23.2"E, 518 m a.s.l., 14.07.2015, all leg. P.N.; 3 juv. (ASU), foot of S slope of mountain, *Padus avium* and *Populus tremula* stand near brook, hand sampling, 31.05.2016; 12 juv. (ASU), site 1 on S slope, soil sample 1 (0–10 cm deep), 31.05.2016; 2 juv. (ASU), site 1 on S slope, soil sample 2 (0–10 cm deep), 31.05.2016; 2 juv. (ASU), site 1 on S slope, soil sample 3 (0–10 cm deep), 31.05.2016; 5 juv. (ASU), site 1 on S slope, soil sample 4 (0–10 cm deep), 1.06.2016; 5 juv. (ASU), site 1 on S slope, soil sample 5 (0–10 cm deep), 1.06.2016; 1 juv. (ASU), S slope between site 1 and site 2, broad gully with *Padus avium*, hand sampling, 1.06.2016; 43 juv. (ASU), site 1 on S slope, hand sampling, 1.06.2016; 9 juv. (ASU), site 2 on S slope, soil sample 1 (0–10 cm deep), 1.06.2016; 11 juv. (ASU), site 2 on S slope, soil sample 2 (0–10 cm deep), 1.06.2016; 4 juv. (ASU), site 2 on S slope, soil sample 3 (0–10 cm deep), 1.06.2016; 2 juv. (ASU), site 2 on S slope, soil sample 4 (0–10 cm deep), 1.06.2016; 3 juv. (ASU), site 2 on S slope, soil sample 5 (0–10 cm deep), 1.06.2016; 4 juv. (ASU), site 2 on S slope, hand sampling, 1.06.2016; 3 juv. (ASU), site 2 on N slope, soil sample 2 (0–10 cm deep), 2.06.2016; 3 juv. (ASU), site 2 on N slope, hand sampling, 2.06.2016, all leg. P.N., Kh.N., S.N., V.S.; 1 ♀ (ASU), *Betula pendula* and *Populus tremula* stand on N slope, 51°21'33.8"N, 83°37'23.2"E, 518 m a.s.l., 12.07.2016, leg. P.N.; 1 ♂, 6 juv., 1 fragm. (ASU), site 1 on S slope, soil sample 1 (0–10 cm deep), 12.07.2016; 2 juv., 1 fragm. (ASU), site 1 on S slope, soil sample 1 (10–20 cm deep), 12.07.2016; 3 juv. (ASU), site 1 on S slope, soil sample 2 (0–10 cm deep), 12.07.2016; 1 ♂, 1 ♀ (ASU), site 1 on S slope, soil sample 2 (10–20 cm deep), 12.07.2016; 1 ♀, 3 juv. (ASU), site 1 on S slope, soil sample 3 (0–10 cm deep), 12.07.2016; 1 ♀, 2 juv. (ASU), site 1 on S slope, soil sample 3 (10–20 cm deep), 12.07.2016; 3 ♀♀, 8 juv. (ASU), site 1 on S slope, soil sample 5 (0–10 cm deep), 12.07.2016; 11 juv. (ASU), site 1 on S slope, hand sampling, 12.07.2016; 1 ♀, 1 juv. (ASU), site 2 on S slope, soil sample 2 (0–10 cm deep), 12.07.2016; 1 juv. (ASU), site 2 on S slope, soil sample 4 (0–10 cm deep), 12.07.2016; 1 ♂, 1 ♀ (ASU), site 2 on S slope, soil sample 5 (0–10 cm deep), 12.07.2016; 1 ♂, 1 fragm. (ASU), site 2 on N slope, soil sample 1 (0–10 cm deep), 13.07.2016; 1 juv. (ASU), site 2 on N slope, soil sample 1 (10–20 cm deep), 13.07.2016; 1 ♂ (ASU), site 2 on N slope, soil sample 2 (litter), 13.07.2016; 1 ♀, 2 juv. (ASU), site 2 on N slope, soil sample 2 (0–10 cm deep), 13.07.2016; 2 juv. (ASU), site 2 on N slope, soil sample 3 (0–10 cm deep), 13.07.2016; 1 ♂ (ASU), site 2 on N slope, soil sample 4 (litter), 13.07.2016; 1 juv. (ASU), site 2 on N slope, soil sample 4 (0–10 cm deep), 13.07.2016, all leg. Kh.N., S.N., V.S.; 2 juv. (ASU), site 1 on S slope, soil sample 1 (0–10 cm deep), 22.08.2016; 2 juv. (ASU), site 1 on S slope, soil sample 2 (0–10 cm deep), 22.08.2016; 2 juv. (ASU), site 1 on S slope, soil sample 3 (0–10 cm deep), 22.08.2016; 2 ♀♀, 3 juv. (ASU), site 1 on S slope, soil sample 4 (0–10 cm deep), 22.08.2016; 4 juv. (ASU), site 1 on S slope, soil sample 5 (0–10 cm deep), 22.08.2016; 1 ♂, 2 ♀♀, 3 juv. (ASU), site 2 on S slope, soil sample 2 (0–10 cm deep), 22.08.2016; 1 ♂, 1 ♀, 5 juv. (ASU), site 2 on S slope, soil sample 3 (0–10 cm deep), 22.08.2016; 2 ♀♀ (ASU), site 2 on S slope, soil sample 4 (0–10 cm deep), 23.08.2016; 1 ♀, 1 juv. (ASU), site 2 on S slope, soil sample 5 (0–10 cm deep), 23.08.2016; 1 ♀, 1 juv. (ASU), site 2 on N slope, soil sample 1 (0–10 cm deep), 23.08.2016; 1 juv., 1 fragm. (ASU), site 2 on N slope, soil sample 4 (0–10 cm deep), 23.08.2016; 1 ♂ (ASU), site 2 on N slope, soil sample 5 (0–10 cm deep), 23.08.2016; 1 ♀ (ASU), site 2 on N slope, hand sampling, 23.08.2016, all leg. P.N., Kh.N., S.N., V.S.; 1 ♀, 1 fragm. (ASU), *Betula pendula* and *Populus tremula* stand on N slope, 51°21'33.8"N, 83°37'23.2"E, 518 m a.s.l., hand sampling, 20.06.2017; 1 juv. (ASU), site 2 on S slope, hand sampling, 24.06.2017, all leg. P.N.

######## Distribution.

European–Western Siberian temperate range: this species appears to be widespread from northern and central Europe (Scandinavia, Finland, the Baltics, Germany, Poland, Belarus) through European Russia and the Urals to East Kazakhstan and SW Siberia (Altai Province, Republic of Altai and Novosibirsk Area).

######## Remarks.

In the study area, *M. sjaelandicum* dominates habitats on the southern slope, where its abundance reaches up to 22 ind./m^2^.

###### Genus *Sibiriulus* Gulička, 1963

####### Sibiriulus latisupremus

Taxon classificationAnimaliaJulidaJulidae

Mikhaljova, Nefediev & Nefedieva, 2014

Figure 7

Sibiriulus multinicus pro parte – Mikhaljova and Nefediev 2003: 85, figs 1–3; Mikhaljova et al. 2007: 60, 61: figs 12–14, 18.
Sibiriulus latisupremus Mikhaljova, Nefediev & Nefedieva, 2014: 35, 36–38: figs, 51: map.
Sibiriulus latisupremus – Mikhaljova 2017: 90, 91: figs, insets 741, 743, 748, 752, 753, 785, 786, 92: map; Nefediev et al. 2017c: 13.

######## Material examined

(all from Russia, southwestern Siberia, Altai Province, Charysh District, ca. 4.5 km SE of Charyshskoye Village). 1 ♂, 3 ♀♀, 1 juv. (ASU), site 1 on S slope, 13.07.2015; 3 ♀♀ (ASU), site 1 on S slope, 13.07.2015, all leg. P.N.; 1 ♂ (ASU), foot of S slope of mountain, *Padus avium* and *Populus tremula* stand near brook, hand sampling, 31.05.2016; 4 ♀♀ (ASU), site 1 on S slope, soil sample 1 (0–10 cm deep), 31.05.2016; 2 ♂♂, 2 ♀♀, 2 juv. (ASU), site 1 on S slope, soil sample 2 (0–10 cm deep), 31.05.2016; 2 ♀♀, 1 fragm. (ASU), site 1 on S slope, soil sample 3 (0–10 cm deep), 31.05.2016; 1 ♀, 1 fragm. (ASU), site 1 on S slope, soil sample 3 (10–20 cm deep), 31.05.2016; 3 ♂♂, 2 ♀♀, 1 juv. (ASU), site 1 on S slope, soil sample 4 (0–10 cm deep), 1.06.2016; 2 ♂♂, 1 ♀ (ASU), site 1 on S slope, soil sample 4 (10–20 cm deep), 1.06.2016; 3 ♀♀, 2 juv., 1 fragm. (ASU), site 1 on S slope, soil sample 5 (0–10 cm deep), 1.06.2016; 6 ♂♂, 9 ♀♀, 3 juv. (ASU), site 1 on S slope, hand sampling, 1.06.2016; 2 juv. (ASU), site 2 on S slope, soil sample 1 (0–10 cm deep), 1.06.2016; 1 ♀ (ASU), site 2 on S slope, soil sample 2 (0–10 cm deep), 1.06.2016; 1 juv. (ASU), site 2 on S slope, soil sample 3 (0–10 cm deep), 1.06.2016; 1 ♂, 6 ♀♀, 2 juv. (ASU), site 2 on S slope, soil sample 4 (0–10 cm deep), 1.06.2016; 3 ♀♀, 1 juv. (ASU), site 2 on S slope, hand sampling, 1.06.2016; 1 ♀, 1 juv., 1 fragm. (ASU), site 1 on N slope, soil sample 1 (litter), 2.06.2016; 1 ♂, 4 ♀♀, 4 juv. (ASU), site 1 on N slope, soil sample 1 (0–10 cm deep), 2.06.2016; 1 fragm. (ASU), site 1 on N slope, soil sample 2 (litter), 2.06.2016; 2 ♂♂, 3 ♀♀, 3 juv., 1 fragm. (ASU), site 1 on N slope, soil sample 2 (0–10 cm deep), 2.06.2016; 2 ♀♀ (ASU), site 1 on N slope, soil sample 3 (litter), 2.06.2016; 1 juv. (ASU), site 1 on N slope, soil sample 3 (0–10 cm deep), 2.06.2016; 1 ♀, 1 juv. (ASU), site 1 on N slope, soil sample 4 (litter), 2.06.2016; 1 ♀, 1 fragm. (ASU), site 1 on N slope, soil sample 4 (0–10 cm deep), 2.06.2016; 1 juv. (ASU), site 1 on N slope, soil sample 4 (10–20 cm deep), 2.06.2016; 1 juv. (ASU), site 1 on N slope, soil sample 5 (litter), 2.06.2016; 2 ♀♀, 2 juv. (ASU), site 1 on N slope, soil sample 5 (0–10 cm deep), 2.06.2016; 1 ♂, 12 ♀♀ (ASU), site 1 on N slope, hand sampling, 2.06.2016; 1 ♀, 2 juv. (ASU), site 2 on N slope, soil sample 1 (0–10 cm deep), 2.06.2016; 1 ♀ (ASU), site 2 on N slope, soil sample 4 (litter), 2.06.2016; 1 juv. (ASU), site 2 on N slope, soil sample 4 (0–10 cm deep), 2.06.2016, all leg. P.N., Kh.N., S.N., V.S.; 8 ♂♂, 5 ♀♀ (ASU), site 1 on N slope, hand sampling, 22.06.2016, leg. Kh.N.; 2 juv. (ASU), site 1 on S slope, soil sample 1 (0–10 cm deep), 12.07.2016; 1 juv. (ASU), site 1 on S slope, soil sample 1 (10–20 cm deep), 12.07.2016; 1 ♀, 3 juv. (ASU), site 1 on S slope, soil sample 2 (0–10 cm deep), 12.07.2016; 2 juv. (ASU), site 1 on S slope, soil sample 2 (10–20 cm deep), 12.07.2016; 1 juv. (ASU), site 1 on S slope, soil sample 3 (0–10 cm deep), 12.07.2016; 4 juv. (ASU), site 1 on S slope, soil sample 4 (0–10 cm deep), 12.07.2016; 1 ♀, 1 juv. (ASU), site 1 on S slope, soil sample 4 (10–20 cm deep), 12.07.2016; 1 ♂, 1 ♀, 3 juv. (ASU), site 1 on S slope, soil sample 5 (0–10 cm deep), 12.07.2016; 2 ♂♂ (ASU), site 2 on S slope, soil sample 2 (0–10 cm deep), 12.07.2016; 1 ♀ (ASU), site 2 on S slope, soil sample 2 (10–20 cm deep), 12.07.2016; 3 juv. (ASU), site 2 on S slope, soil sample 3 (0–10 cm deep), 12.07.2016; 2 juv. (ASU), site 2 on S slope, soil sample 4 (0–10 cm deep), 12.07.2016; 2 ♂♂, 6 juv. (ASU), site 1 on N slope, soil sample 1 (0–10 cm deep), 13.06.2016; 1 juv. (ASU), site 1 on N slope, soil sample 1 (10–20 cm deep), 13.06.2016; 1 ♂, 4 ♀♀, 2 juv. (ASU), site 1 on N slope, soil sample 2 (0–10 cm deep), 13.06.2016; 2 ♀♀, 1 juv. (ASU), site 1 on N slope, soil sample 3 (0–10 cm deep), 13.06.2016; 1 juv. (ASU), site 1 on N slope, soil sample 4 (0–10 cm deep), 13.06.2016; 1 ♂ (ASU), site 1 on N slope, soil sample 5 (0–10 cm deep), 13.06.2016; 2 ♀♀, 1 juv. (ASU), site 1 on N slope, hand sampling, 13.06.2016; 1 juv. (ASU), site 2 on N slope, soil sample 5 (0–10 cm deep), 13.06.2016, all leg. Kh.N., S.N., V.S.; 2 ♀♀, 1 juv. (ASU), site 1 on S slope, soil sample 1 (0–10 cm deep), 22.08.2016; 1 juv. (ASU), site 1 on S slope, soil sample 2 (0–10 cm deep), 22.08.2016; 3 juv. (ASU), site 1 on S slope, soil sample 3 (0–10 cm deep), 22.08.2016; 2 ♂♂ (ASU), site 1 on S slope, soil sample 3 (10–20 cm deep), 23.08.2016; 1 ♀, 1 juv. (ASU), site 1 on S slope, soil sample 5 (0–10 cm deep), 23.08.2016; 1 ♀ (ASU), site 1 on S slope, soil sample 5 (10–20 cm deep), 23.08.2016; 2 ♀♀, 1 juv., 1 fragm. (ASU), site 1 on N slope, soil sample 1 (0–10 cm deep), 23.08.2016; 2 ♀♀, 1 juv. (ASU), site 1 on N slope, soil sample 2 (0–10 cm deep), 23.08.2016; 2 ♀♀ (ASU), site 1 on N slope, soil sample 5 (0–10 cm deep), 23.08.2016; 1 ♂, 4 ♀♀ (ASU), site 2 on N slope, soil sample 2 (0–10 cm deep), 23.08.2016; 1 ♀ (ASU), site 2 on N slope, hand sampling, 23.08.2016, all leg. P.N., Kh.N., S.N., V.S.; 3 ♂♂, 5 ♀♀, 1 juv. (ZMUM), site 1 on N slope, hand sampling, 23.06.2017, leg. P.N., Kh.N., A.A., E.A.; 1 juv. (ASU), site 2 on S slope, hand sampling, 24.06.2017, leg. P.N.

######## Distribution.

Being an endemic of SW Siberia, *S. latisupremus* has previously been recorded in a few localities in SE part of the Altai Province and NW part of the Republic of Altai (Mikhaljova et al. 2014).

######## Remarks.

The above records of the julid *S. latisupremus* are the southwesternmost for the species. In the study localities, the species demonstrates no preference between investigated habitats as regards different slope exposures.

##### Family Nemasomatidae Bollman, 1893

###### Genus *Orinisobates* Lohmander, 1933

####### Orinisobates sibiricus

Taxon classificationAnimaliaJulidaNemasomatidae

(Gulička, 1963)

Isobates sibiricus Gulička, 1963: 522: figs.
Isobates sibiricus – Byzova and Chadaeva 1965: 337.
Isobates (Orinisobates) sibiricus – Gulička 1972: 45: figs; Nefediev and Nefedieva 2008a: 117; Babenko et al. 2009: 182.
Orinisobates sibiricus – Enghoff 1985: 53, 54: figs; Mikhaljova 1993: 16; 2002: 206; 2004: 96: figs, 94: map; 2017: 120, 121: figs, 122: map; Mikhaljova and Golovatch 2001: 107; Mikhaljova and Nefediev 2003: 83; Nefediev and Nefedieva 2006: 98; 2007a: 139; 2007b: 160; 2008a: 117; 2008b: 62; 2013: 87; Nefedieva and Nefediev 2008: 123; Nefediev et al. 2014a: 63; 2017c: 13; Nefedieva et al. 2014: 65; 2015: 147.

######## Material examined

(all from Russia, southwestern Siberia, Altai Province, Charysh District, ca. 4.5 km SE of Charyshskoye Village). 1 ♀ (ASU), site 1 on S slope, 13.07.2015, leg. P.N.; 1 ♀ (ASU), site 2 on S slope, soil sample 1 (0–10 cm deep), 1.06.2016; 1 ♂, 1 ♀, 1 juv. (ASU), site 2 on S slope, soil sample 4 (0–10 cm deep), 1.06.2016; 1 ♂ (ASU), site 1 on N slope, soil sample 1 (litter), 2.06.2016; 3 ♂♂, 1 juv. (ASU), site 1 on N slope, soil sample 1 (0–10 cm deep), 2.06.2016; 1 ♀ (ASU), site 1 on N slope, soil sample 2 (0–10 cm deep), 2.06.2016; 6 ♀♀ (ASU), site 1 on N slope, hand sampling, 2.06.2016, all leg. P.N., Kh.N., S.N., V.S.; 2 ♀♀ (ASU), site 1 on N slope, hand sampling, 22.06.2016, leg. Kh.N.; 1 ♂, 1 ♀, 4 juv. (ASU), site 2 on S slope, soil sample 2 (0–10 cm deep), 12.07.2016; 1 ♀, 1 juv., 1 fragm. (ASU), site 2 on S slope, soil sample 2 (10–20 cm deep), 12.07.2016; 1 ♂, 1 ♀ (ZMUM), 2 ♂♂, 3 juv. (ASU), site 1 on N slope, soil sample 1 (0–10 cm deep), 13.07.2016; 2 ♂♂, 1 ♀, 1 juv. (ASU), site 2 on N slope, soil sample 1 (0–10 cm deep), 13.07.2016, all leg. Kh.N., S.N., V.S.; 1 ♂ (ASU), site 2 on S slope, soil sample 1 (0–10 cm deep), 22.08.2016, leg. P.N., Kh.N., S.N., V.S.

######## Distribution.

Being a Central Palaearctic species, *O. sibiricus* is very widespread in southern Siberia, Russia as far as the Zabaikalskii Province, Republic of Tyva, southern part of the Krasnoyarsk Province, Republic of Khakassia, Republic of Altai, Altai Province and Kemerovo Area; also known from Eastern Kazakhstan and Kyrgyzstan.

######## Remarks.

This species shows no significant difference in its abundance between two studied slope exposures.

#### Order Chordeumatida C. L. Koch, 1847

##### Family Diplomaragnidae Attems, 1907

###### Genus *Altajosoma* Gulička, 1972

####### Altajosoma

Taxon classificationAnimaliaChordeumatidaDiplomaragnidae

sp.

######## Material examined

(all from Russia, southwestern Siberia, Altai Province, Charysh District, ca. 4.5 km SE of Charyshskoye Village). 1 ♂, 1 ♀ (ASU), site 1 on N slope, 13.07.2015; 2 juv. (ASU), *Betula pendula* and *Populus tremula* stand on N slope, 51°21'33.8"N, 83°37'23.2"E, 518 m a.s.l., 14.07.2015, all leg. P.N.; 1 juv. (ASU), site 1 on S slope, soil sample 5 (0–10 cm deep), 1.06.2016; 1 juv. (ASU), site 1 on N slope, soil sample 1 (litter), 2.06.2016; 1 juv. (ASU), site 1 on N slope, soil sample 2 (litter), 2.06.2016; 1 juv. (ASU), site 1 on N slope, soil sample 4 (litter), 2.06.2016; 1 juv. (ASU), site 1 on N slope, hand sampling, 2.06.2016, all leg. P.N., Kh.N., S.N., V.S.; 2 juv. (ASU), site 1 on N slope, hand sampling, 22.06.2016, leg. Kh.N.; 1 ♂, 1 ♀ (ASU), site 1 on S slope, soil sample 2 (0–10 cm deep), 12.07.2016; 1 ♀ (ASU), site 1 on S slope, soil sample 4 (0–10 cm deep), 12.07.2016; 1 ♀ (ASU), site 1 on S slope, soil sample 4 (10–20 cm deep), 12.07.2016; 1 ♀ (ASU), site 1 on N slope, soil sample 2 (0–10 cm deep), 13.07.2016; 1 ♀, 1 juv. (ASU), site 2 on N slope, soil sample 5 (0–10 cm deep), 13.07.2016, all leg. Kh.N., S.N., V.S.; 1 juv. (ASU), site 2 on S slope, soil sample 2 (litter), 22.07.2016; 1 ♂ (ASU), site 1 on N slope, soil sample 2 (0–10 cm deep), 23.08.2016; 1 juv. (ASU), site 2 on N slope, soil sample 5 (0–10 cm deep), 23.08.2016, all leg. P.N., Kh.N., S.N., V.S.; 4 juv. (ASU), site 1 on N slope, hand sampling, 23.06.2017, leg. P.N., Kh.N., A.A., E.A.

######## Distribution.

This species is currently known only from the study area.

######## Remarks.

The above recorded specimens of *Altajosoma* sp. are most similar to *Altajosoma bakurovi bakurovi* (Shear, 1990) in some details of gonopod structure, i.e. in the shape of colpocoxites of the posterior gonopods and in particular in their distal parts, but the colpocoxites are a little bit narrower in the newly found species compared to *A. bakurovi bakurovi*. These specimens also differ significantly in the shape of the large posterior angiocoxal processes.

#### Order Polydesmida Leach, 1815

##### Family Polydesmidae Leach, 1815

###### Genus *Schizoturanius* Verhoeff, 1931

####### Schizoturanius clavatipes

Taxon classificationAnimaliaPolydesmidaPolydesmidae

(Stuxberg, 1876)

Polydesmus clavatipes – Nefediev and Nefedieva 2008a: 117.
Schizoturanius clavatipes – Mikhaljova 1993: 31, 32: figs; 2002: 206; 2004: 238, 239: figs, 228: map; 2013: 9; 2016: 24; 2017: 288, 289: figs, 290: map; Nefediev 2001: 85; 2002a: 30; 2002b: 139; Mikhaljova and Golovatch 2001: 116; Mikhaljova and Nefediev 2003: 81; Nefediev and Nefedieva 2005: 178; 2006: 98; 2007a: 139; 2007b: 161;2007c: 99; 2008b: 62; 2011: 100; 2012a: 51; 2012b: 47; 2013: 87; Nefedieva and Nefediev 2008: 123; Nefediev et al. 2014a: 63; 2017c: 13; Nefedieva et al. 2014: 65; 2015: 152.

######## Material examined

(all from Russia, southwestern Siberia, Altai Province, Charysh District, ca. 4.5 km SE of Charyshskoye Village). 2 juv. (ASU), *Betula pendula* and *Populus tremula* stand on N slope, 51°21'33.8"N, 83°37'23.2"E, 518 m a.s.l., 14.07.2015, leg. P.N.; 4 juv. (ASU), site 1 on S slope, soil sample 3 (0–10 cm deep), 1.06.2016; 2 ♀♀, 9 juv. (ASU), site 1 on S slope, soil sample 4 (0–10 cm deep), 1.06.2016; 1 juv. (ASU), site 1 on S slope, soil sample 5 (0–10 cm deep), 1.06.2016; 2 ♂♂, 1 ♀, 2 juv. (ASU), foot of S slope of mountain, *Padus avium* and *Populus tremula* stand near brook, hand sampling, 1.06.2016; 2 ♂♂, 2 ♀♀ (ASU), site 1 on N slope, hand sampling, 2.06.2016, all leg. P.N., Kh.N., S.N., V.S.; 2 juv. (ASU), site 1 on S slope, soil sample 1 (0–10 cm deep), 12.07.2016; 1 juv. (ASU), site 1 on S slope, soil sample 3 (0–10 cm deep), 12.07.2016; 3 juv. (ASU), site 1 on S slope, soil sample 3 (10–20 cm deep), 12.07.2016; 2 juv. (ASU), site 1 on S slope, soil sample 5 (0–10 cm deep), 12.07.2016; 1 juv. (ASU), site 1 on N slope, soil sample 1 (0–10 cm deep), 13.07.2016; 1 juv. (ASU), site 1 on N slope, hand sampling, 13.07.2016; 1 juv. (ASU), site 2 on N slope, soil sample 3 (0–10 cm deep), 13.07.2016; 1 juv. (ASU), near Komendantka Village, hand sampling, 14.07.2016, all leg. Kh.N., S.N., V.S.; 1 ♂ (ASU), site 1 on S slope, soil sample 2 (10–20 cm deep), 22.08.2016; 1 ♂ (ASU), site 1 on S slope, soil sample 4 (0–10 cm deep), 23.08.2016; 1 ♂, 1 ♀ (ZMUM), 1 ♀ (ASU), site 1 on S slope, soil sample 5 (0–10 cm deep), 23.08.2016; 1 ♂ (ASU), site 2 on S slope, soil sample 2 (0–10 cm deep), 22.08.2016; 1 juv. (ASU), site 2 on S slope, soil sample 4 (0–10 cm deep), 22.08.2016; 1 ♂ (ASU), site 2 on N slope, soil sample 2 (0–10 cm deep), 23.08.2016; 1 ♀ (ASU), site 2 on N slope, soil sample 4 (litter), 23.08.2016, all leg. P.N., Kh.N., S.N., V.S.

######## Distribution.

Being a Western-Central Siberian species, *S. clavatipes* appears to be very widespread in southwestern Siberia, Russia, inhabiting Tomsk, Novosibirsk, and Kemerovo areas, Altai Province, Republic of Altai, Republic of Khakassia, and also along the Yenisei River in the Krasnoyarsk Province, central Siberia, Russia.

######## Remarks.

The results of this study suggest that *S. clavatipes* prefers the southern slope, in spite of its highly ecological valence.

### Class Chilopoda Latreille, 1817

#### Order Lithobiomorpha Pocock, 1895

##### Family Lithobiidae Newport, 1844

###### Genus *Lithobius* Leach, 1814

####### Lithobius (Ezembius) ostiacorum

Taxon classificationAnimaliaLithobiomorphaLithobiidae

Stuxberg, 1876

Lithobius (Ezembius) ostiacorum – Nefediev et al. 2017c: 13; 2017d: 218: map.

######## Material examined

(all from Russia, southwestern Siberia, Altai Province, Charysh District, ca. 4.5 km SE of Charyshskoye Village). 1 ♀ (ZMUM), foot of S slope of mountain, *Padus avium* and *Populus tremula* stand near brook, hand sampling, 31.05.2016; 1 ♀ (ASU), site 1 on N slope, soil sample 1 (0–10 cm deep), 2.06.2016; 1 juv. (ASU), site 1 on N slope, soil sample 2 (0–10 cm deep), 2.06.2016; 1 ♂ (ASU), site 1 on N slope, soil sample 3 (litter), 2.06.2016; 1 ♀, 1 juv. (ASU), site 1 on N slope, soil sample 5 (0–10 cm deep), 2.06.2016, all leg. P.N., Kh.N., S.N., V.S.; 1 ♀ (ASU), site 1 on S slope, soil sample 3 (0–10 cm deep), 12.07.2016; 2 ♀♀, 1 juv. (ASU), site 1 on N slope, soil sample 2 (0–10 cm deep), 12.07.2016; 1 ♂, 1 juv. (ASU), site 1 on N slope, soil sample 5 (0–10 cm deep), 12.07.2016; 1 juv. (ASU), site 2 on N slope, soil sample 2 (0–10 cm deep), 13.07.2016; 1 ♀ (ASU), site 2 on N slope, soil sample 4 (10–20 cm deep), 13.07.2016, all leg. Kh.N., S.N., V.S.; 2 juv. (ASU), site 1 on S slope, soil sample 5 (0–10 cm deep), 22.08.2016; 1 juv. (ASU), site 2 on N slope, soil sample 2 (0–10 cm deep), 23.08.2016; 2 juv. (ASU), site 2 on N slope, soil sample 3 (0–10 cm deep), 23.08.2016, all leg. P.N., Kh.N., S.N., V.S.; 1 ♂ (PSU), *Betula pendula* and *Populus tremula* stand on N slope, 51°21'33.8"N, 83°37'23.2"E, 518 m a.s.l., hand sampling, 20.06.2017, leg. P.N.

######## Distribution.

Southern Siberian boreal range with isolated Yenisei population: this species has previously been recorded in the Yenisei River area, Krasnoyarsk Province and Irkutsk Area (central and eastern Siberia, respectively) (Zalesskaja 1978), also recently found in northern Mongolia (Poloczek et al. 2016), Altai Province (Nefediev et al. 2017c) and Republic of Altai (Nefediev et al. 2017d).

######## Remarks.

The above record of *L. ostiacorum*, recently announced at the 17th International Congress of Myriapodology (Nefediev et al. 2017c), can be considered as the first formal find of the species in SW Siberia. In the study localities, the species was found more frequently on N facing habitats.

####### Lithobius (Ezembius) proximus

Taxon classificationAnimaliaLithobiomorphaLithobiidae

Sseliwanoff, 1880

Lithobius proximus – Zalesskaja 1978: 125–126; Striganova and Poryadina 2005: 226; Bukhkalo and Sergeeva 2012: 61; Sergeeva 2013: 530–532; Bukhkalo et al. 2014: 71–72;
Lithobius (Ezembius) proximus – Nefediev et al. 2017b: 116, 117: map; 2017c: 13; 2017d: 218: map.

######## Material examined

(all from Russia, southwestern Siberia, Altai Province, Charysh District, ca. 4.5 km SE of Charyshskoye Village). 2 ♂♂ (ASU), *Betula pendula* and *Populus tremula* stand on N slope, 51°21'33.8"N, 83°37'23.2"E, 518 m a.s.l., 14.07.2015, leg. P.N.; 4 ♂♂ (ASU), same locality, 15.07.2015, leg. P.N., T.Z.; 1 ♂ (ASU), S slope between site 1 and site 2, broad gully with *Padus avium*, hand sampling, 31.05.2016, leg. P.N., Kh.N., S.N., V.S.; 1 subadult ♂ (ASU), *Betula pendula* and *Populus tremula* stand on N slope, 51°21'33.8"N, 83°37'23.2"E, 518 m a.s.l., 12.07.2016; 2 ♂♂, 1 ♀ (ASU), same locality, pitfall traps, 12–14.07.2016, all leg. P.N.; 1 ♂ (ASU), site 2 on S slope, soil sample 1 (0–10 cm deep), 12.07.2016, leg. Kh.N., S.N., V.S.; 2 juv. (ASU), site 1 on N slope, soil sample 2 (0–10 cm deep), 23.08.2016; 1 ♂ (ASU), site 2 on N slope, soil sample 4 (litter), 23.08.2016; 1 ♀ (ASU), site 2 on N slope, hand sampling, 23.08.2016, all leg. P.N., Kh.N., S.N., V.S.; 1 ♂ (ASU), *Betula pendula* and *Populus tremula* stand on N slope, 51°21'33.8"N, 83°37'23.2"E, 518 m a.s.l., hand sampling, 20.06.2017, leg. P.N.

######## Distribution.

Eastern European-transSiberian temperate range: this species is widespread from the eastern Russian Plain (republics of Mari El and Tatarstan, Kirov and Samara areas) in the west through Siberia to the Russian Far East (Maritime Province, Sakhalin and the Kuriles) (Zalesskaja 1978; Farzalieva and Esyunin 2008; Farzalieva 2009; Farzalieva and Tselishcheva 2009).

######## Remarks.

The above find of the species, recently announced at the 17th International Congress of Myriapodology (Nefediev et al. 2017c), can be considered as the first formal record of it in the Altai Province, SW Siberia. In the investigated area, *L. proximus* is very rare and shows no significant differences in its distribution between slopes.

####### Lithobius (Ezembius) sibiricus

Taxon classificationAnimaliaLithobiomorphaLithobiidae

Gerstfeldt, 1858

Lithobius sibiricus – Nefediev 2001: 85.
Lithobius (Ezembius) sibiricus – Nefediev et al. 2016d: 263; 2017c: 13; 2017d: 219, 218: map.

######## Material examined

(all from Russia, southwestern Siberia, Altai Province, Charysh District, ca. 4.5 km SE of Charyshskoye Village). 2 ♂♂, 1 ♀, 2 juv. (ASU), site 1 on S slope, 13.07.2015; 1 ♂, 1 ♀, 2 subadult ♀♀ (ASU), *Betula pendula* and *Populus tremula* stand on N slope, 51°21'33.8"N, 83°37'23.2"E, 518 m a.s.l., 14.07.2015, all leg. P.N.; 1 ♂, 1 subadult ♀, 2 juv. (ZMUM), foot of S slope of mountain, *Padus avium* and *Populus tremula* stand near brook, hand sampling, 31.05.2016; 1 ♂, 1 ♀, 1 juv. (PSU), site 1 on S slope, hand sampling, 31.05.2016; 2 ♂♂, 8 ♀♀ (ASU), S slope between site 1 and site 2, broad gully with *Padus avium*, hand sampling, 1.06.2016; 1 ♂ (ASU), site 1 on S slope, soil sample 3 (10–20 cm deep), 1.06.2016; 2 juv. (ASU), site 1 on N slope, soil sample 2 (0–10 cm deep), 2.06.2016; 7 ♂♂, 1 ♀, 3 juv. (ASU), site 1 on N slope, soil sample 3 (litter), 2.06.2016; 1 ♂ (ASU), site 1 on N slope, soil sample 3 (10–20 cm deep), 1.06.2016; 1 ♀ (ASU), site 1 on N slope, soil sample 4 (0–10 cm deep), 2.06.2016; 2 ♂♂, 1 ♀ (ASU), site 1 on N slope, hand sampling, 2.06.2016; 1 ♂, 1 subadult ♂, 4 ♀♀, 1 subadult ♀ (ASU), site 2 on N slope, hand sampling, 2.06.2016; 1 juv. (ASU), site 2 on N slope, soil sample 1 (0–10 cm deep), 2.06.2016; 1 juv. (ASU), site 2 on N slope, soil sample 1 (10–20 cm deep), 2.06.2016, all leg. P.N., Kh.N., S.N., V.S.; 4 ♂♂, 1 ♀ (ASU), *Betula pendula* and *Populus tremula* stand on N slope, 51°21'33.8"N, 83°37'23.2"E, 518 m a.s.l., pitfall traps, 12–14.07.2016, leg. P.N.; 1 ♀ (ASU), site 1 on S slope, soil sample 1 (10–20 cm deep), 12.07.2016; 1 ♂, 3 juv. (ASU), site 2 on S slope, soil sample 1 (0–10 cm deep), 12.07.2016; 1 ♂, 2 ♀♀, 1 juv. (ASU), site 2 on S slope, soil sample 1 (10–20 cm deep), 12.07.2016; 1 ♀ (ASU), site 2 on S slope, soil sample 2 (10–20 cm deep), 12.07.2016; 1 ♂ (ASU), site 1 on N slope, soil sample 1 (10–20 cm deep), 13.07.2016; 2 ♂♂, 1 ♀, 1 juv. (ASU), site 1 on N slope, soil sample 2 (0–10 cm deep), 13.07.2016; 1 juv. (ASU), site 1 on N slope, soil sample 5 (0–10 cm deep); 1 ♂ (ASU), site 2 on N slope, hand sampling, 13.07.2016; 1 ♂ (ASU), site 2 on N slope, soil sample 1 (0–10 cm deep), 13.07.2016; 1 ♂, 1 ♀ (ASU), site 2 on N slope, soil sample 3 (0–10 cm deep), 13.07.2016; 1 ♀ (ASU), site 2 on N slope, soil sample 4 (litter), 13.07.2016; 1 ♂, 1 fragm. (ASU), site 2 on N slope, soil sample 4 (0–10 cm deep), 13.07.2016; 1 ♂ (ASU), near Komendantka Village, hand sampling, 14.07.2016, all leg. Kh.N., S.N., V.S.; 1 juv. (ASU), site 1 on S slope, soil sample 4 (0–10 cm deep), 23.08.2016; 1 ♀, 1 juv. (ASU), site 1 on S slope, soil sample 5 (0–10 cm deep), 23.08.2016; 1 ♂, 1 ♀ (ASU), site 1 on N slope, soil sample 1 (0–10 cm deep), 23.08.2016; 1 ♂, 1 juv., 1 fragm. (ASU), site 1 on N slope, soil sample 2 (0–10 cm deep), 23.08.2016; 1 juv. (ASU), site 1 on N slope, soil sample 3 (0–10 cm deep), 23.08.2016; 1 ♂ (ASU), site 1 on N slope, soil sample 4 (0–10 cm deep), 23.08.2016; 1 ♀ (ASU), site 2 on N slope, soil sample 2 (0–10 cm deep), 23.08.2016; 1 ♀ (ASU), site 2 on N slope, soil sample 3 (0–10 cm deep), 23.08.2016; 3 ♂♂, 1 juv. (ASU), site 2 on N slope, soil sample 4 (0–10 cm deep), 23.08.2016; 1 ♂ (ASU), site 2 on N slope, soil sample 5 (litter), 23.08.2016; 2 ♂♂, 1 ♀, 1 juv. (ASU), site 2 on N slope, soil sample 5 (0–10 cm deep), 23.08.2016; 2 ♂♂, 2 ♀♀, 1 juv. (ASU), site 2 on N slope, soil sample, hand sampling, 23.08.2016, all leg. P.N., Kh.N., S.N., V.S.; 1 ♀, 1 juv. (ASU), *Betula pendula* and *Populus tremula* stand on N slope, 51°21'33.8"N, 83°37'23.2"E, 518 m a.s.l., hand sampling, 20.06.2017, leg. P.N.; 2 ♂♂, 3 ♀♀ (ASU), site 1 on N slope, hand sampling, 23.06.2017; 1 ♂ (ASU), site 2 on N slope, hand sampling, 23.06.2017, all leg. P.N., Kh.N., A.A., E.A.

######## Distribution.

Trans-Siberian temperate range: *L. sibiricus* is one of the most widely spread lithobiomorph centipedes in the Asian part of Russia, having been reported from southwestern Siberia (Tomsk Area, Altai Province and Republic of Altai), central and eastern Siberia (Krasnoyarsk Province, Irkutsk Area, Zabaikalskii Province and the republics of Buryatia and Sakha) and the Russian Far East (Amur Area, Maritime Province and Sakhalin Island); also recorded in northern Mongolia (Nefediev et al. 2016, 2017c, d).

######## Remarks.

In the study localities, *L. sibiricus* shows a higher abundance on the northern slope.

####### Lithobius (Monotarsobius) curtipes

Taxon classificationAnimaliaLithobiomorphaLithobiidae

C.L. Koch, 1847

Lithobius curtipes – Striganova and Poryadina 2005: 226; Bukhkalo and Sergeeva 2012: 61; Sergeeva 2013: 530–532.
Lithobius (Monotarsobius) curtipes – Nefediev et al. 2016d: 263, 260: map; 2017b: 116, 117: map; 2017c: 13; 2017d: 219, 218: map.

######## Material examined

(all from Russia, southwestern Siberia, Altai Province, Charysh District, ca. 4.5 km SE of Charyshskoye Village). 1 subadult ♀ (ASU), site 1 on S slope, 13.07.2015, leg. P.N.; 1 ♀ (ZMUM), foot of S slope, *Padus avium* and *Populus tremula* stand near brook, hand sampling, 31.05.2016; 1 ♂ (ASU), site 1 on S slope, soil sample 5 (0–10 cm deep), 1.06.2016; 1 ♂ (ASU), site 1 on N slope, soil sample 4 (litter), 2.06.2016; 1 ♂, 1 juv. (ASU), site 1 on N slope, soil sample 5 (litter), 2.06.2016; 2 ♂♂, 1 juv. (ASU), site 1 on N slope, soil sample 5 (0–10 cm deep), 2.06.2016; 1 ♂, 1 juv. (ASU), site 2 on N slope, soil sample 1 (0–10 cm deep), 2.06.2016; 1 ♂, 2 ♀♀, 2 juv. (ASU), site 2 on N slope, soil sample 2 (0–10 cm deep), 2.06.2016; 1 ♀ (ASU), site 2 on N slope, soil sample 3 (litter), 2.06.2016; 1 ♀, 2 juv. (ASU), site 2 on N slope, soil sample 3 (0–10 cm deep), 2.06.2016, all leg. P.N., Kh.N., S.N., V.S.; 1 ♂, 2 ♀♀ (ASU), *Betula pendula* and *Populus tremula* stand on N slope, 51°21'33.8"N, 83°37'23.2"E, 518 m a.s.l., 12.07.2016, leg. P.N.; 2 ♂♂ (ASU), site 1 on N slope, soil sample 3 (litter), 13.07.2016; 1 ♂ (ASU), site 1 on N slope, soil sample 3 (0–10 cm deep), 13.07.2016; 1 ♂, 1 ♀ (ASU), site 1 on N slope, soil sample 4 (0–10 cm deep), 13.07.2016; 2 ♂♂ (ASU), site 1 on N slope, soil sample 5 (0–10 cm deep), 13.07.2016; 1 ♀ (ASU), site 2 on N slope, soil sample 1 (0–10 cm deep), 13.07.2016; 2 ♂♂ (ASU), site 2 on N slope, soil sample 2 (litter), 13.07.2016; 1 ♂, 4 ♀♀, 2 juv. (ASU), site 2 on N slope, soil sample 3 (0–10 cm deep), 13.07.2016; 1 juv. (ASU), site 2 on N slope, soil sample 4 (litter), 13.07.2016; 1 ♂, 1 ♀ (ASU), site 2 on N slope, soil sample 4 (0–10 cm deep), 13.07.2016, all leg. Kh.N., S.N., V.S.; 2 ♂♂, 2 ♀♀, 1 juv. (ASU), site 2 on N slope, soil sample 1 (0–10 cm deep), 23.08.2016; 1 ♀ (ASU), site 2 on N slope, soil sample 2 (0–10 cm deep), 23.08.2016; 1 ♂, 5 ♀♀ (ASU), site 2 on N slope, soil sample 3 (0–10 cm deep), 23.08.2016; 1 ♀ (ASU), site 2 on N slope, soil sample 3 (10–20 cm deep), 23.08.2016; 1 juv. (ASU), site 2 on N slope, soil sample 4 (litter), 23.08.2016; 2 ♂♂, 2 ♀♀ (ASU), site 2 on N slope, soil sample 4 (0–10 cm deep), 23.08.2016; 6 ♂♂, 2 juv. (ASU), site 2 on N slope, soil sample 5 (0–10 cm deep), 23.08.2016, all leg. P.N., Kh.N., S.N., V.S.; 4 ♂♂, 3 ♀♀ (ASU), *Betula pendula* and *Populus tremula* stand on N slope, 51°21'33.8"N, 83°37'23.2"E, 518 m a.s.l., hand sampling, 20.06.2017, leg. P.N.; 1 subadult ♀ (ASU), site 2 on N slope, hand sampling, 23.06.2017, leg. P.N., Kh.N., A.A., E.A.

######## Distribution.

Trans-Palaearctic: the species displays extremely wide distribution in Europe, Asian Russia, the Near East and the Arabian Peninsula, also in northern Mongolia. In Siberia *L. curtipes* has been reported from the Novosibirsk, Omsk, Tyumen and Tomsk areas, the Altai and Krasnoyarsk provinces and the Republic of Altai (Nefediev et al. 2016d, 2017b, c).

######## Remarks.

Despite a wide geographical range, and its high ecological valence, in the study area, the species inhabits mainly the northern slope.

####### Lithobius (Monotarsobius) insolens

Taxon classificationAnimaliaLithobiomorphaLithobiidae

Dányi & Tuf, 2012

Lithobius (Monotarsobius) insolens – Nefediev et al. 2017b: 116, 117: map; 2017c: 13; 2017d: 221, 220: map.

######## Material examined

(all from Russia, southwestern Siberia, Altai Province, Charysh District, ca. 4.5 km SE of Charyshskoye Village). 1 ♀ (ASU), site 1 on S slope, 13.07.2015; 5 ♂♂, 4 ♀♀, 2 juv. (ASU), *Betula pendula* and *Populus tremula* stand on N slope, 51°21'33.8"N, 83°37'23.2"E, 518 m a.s.l., 14.07.2015, all leg. P.N.; 10 ♂♂, 7 ♀♀, 3 subadult ♀♀, 1 juv. (PSU), site 1 on S slope, hand sampling, 31.05.2016; 1 juv. (ASU), site 1 on S slope, soil sample 1 (10–20 cm deep), 31.05.2016; 1 juv. (ASU), site 1 on S slope, soil sample 2 (0–10 cm deep), 31.05.2016; 2 ♂♂, 1 ♀, 2 juv. (ASU), site 1 on S slope, soil sample 4 (0–10 cm deep), 1.06.2016; 1 ♂, 2 ♀♀ (ASU), site 1 on S slope, soil sample 5 (0–10 cm deep), 1.06.2016; 1 ♂ (ASU), S slope between site 1 and site 2, broad gully with *Padus avium*, hand sampling, 1.06.2016; 1 ♀ (ASU), site 2 on S slope, soil sample 2 (0–10 cm deep), 1.06.2016; 2 ♂♂, 1 ♀, 1 juv. (ASU), site 2 on S slope, soil sample 4 (0–10 cm deep), 1.06.2016; 2 ♀♀ (ASU), site 2 on S slope, soil sample 5 (0–10 cm deep), 1.06.2016; 1 ♂, 2 ♀♀, (ASU), site 2 on S slope, hand sampling, 1.06.2016; 1 ♀ (ASU), site 1 on N slope, soil sample 1 (0–10 cm deep), 2.06.2016; 1 ♂ (ASU), site 1 on N slope, soil sample 2 (0–10 cm deep), 2.06.2016; 1 ♂, 1 ♀ (ASU), site 1 on N slope, soil sample 5 (litter), 2.06.2016; 1 ♂ (ASU), site 1 on N slope, soil sample 5 (0–10 cm deep), 2.06.2016; 3 ♂♂ (ASU), site 1 on N slope, hand sampling, 2.06.2016; 1 ♀ (ASU), site 2 on N slope, soil sample 1 (0–10 cm deep), 2.06.2016; 1 ♀ (ASU), site 2 on N slope, soil sample 4 (litter), 2.06.2016, all leg. P.N., Kh.N., S.N., V.S.; 3 ♂♂, 1 subadult ♂ (ASU), *Betula pendula* and *Populus tremula* stand on N slope, 51°21'33.8"N, 83°37'23.2"E, 518 m a.s.l., 12.07.2016; 1 ♂ (ASU), same locality, pitfall traps, 12–14.07.2016, all leg. P.N.; 1 ♂, 4 ♀♀, 8 juv. (ASU), site 1 on S slope, soil sample 1 (0–10 cm deep), 12.07.2016; 1 ♂, 1 juv. (ASU), site 1 on S slope, soil sample 1 (10–20 cm deep), 12.07.2016; 1 juv. (ASU), site 1 on S slope, soil sample 1 (20–30 cm deep), 12.07.2016; 2 juv. (ASU), site 1 on S slope, soil sample 2 (0–10 cm deep), 12.07.2016; 1 ♀ (ASU), site 1 on S slope, soil sample 3 (0–10 cm deep), 12.07.2016; 3 ♂♂, 2 ♀♀ (ASU), site 1 on S slope, soil sample 4 (0–10 cm deep), 12.07.2016; 2 ♂♂, 1 ♀ (ASU), site 1 on S slope, soil sample 5 (0–10 cm deep), 12.07.2016; 1 ♀, 1 juv. (ASU), site 1 on S slope, hand sampling, 12.07.2016; 2 juv. (ASU), site 2 on S slope, soil sample 2 (0–10 cm deep), 12.07.2016; 2 juv. (ASU), site 2 on S slope, soil sample 5 (0–10 cm deep), 12.07.2016; 2 ♂♂, 1 ♀, 1 juv. (ASU), site 1 on N slope, soil sample 1 (0–10 cm deep), 13.07.2016; 1 ♂, 1 ♀, 2 juv. (ASU), site 1 on N slope, soil sample 3 (0–10 cm deep), 13.07.2016; 2 juv. (ASU), site 1 on N slope, soil sample 4 (0–10 cm deep), 13.07.2016; 1 ♂, 1 juv. (ASU), site 1 on N slope, soil sample 5 (0–10 cm deep), 13.07.2016; 1 ♂, 1 ♀, 2 juv. (ASU), site 2 on N slope, soil sample 1 (0–10 cm deep), 13. 07.2016, all leg. Kh.N., S.N., V.S.; 1 ♂, 3 ♀♀, 1 juv. (ASU), site 1 on S slope, soil sample 1 (0–10 cm deep), 22.08.2016; 2 ♂♂, 6 ♀♀, 6 juv., 1 fragm. (ASU), site 1 on S slope, soil sample 2 (0–10 cm deep), 22.08.2016; 1 ♂, 1 ♀, 1 juv. (ASU), site 1 on S slope, soil sample 3 (0–10 cm deep), 22.08.2016; 4 ♀♀, 5 juv., 1 fragm. (ASU), site 1 on S slope, soil sample 4 (0–10 cm deep), 23.08.2016; 2 ♂♂, 4 juv. (ASU), site 1 on S slope, soil sample 5 (0–10 cm deep), 23.08.2016; 1 ♂ (ASU), site 2 on S slope, soil sample 2 (litter), 22.08.2016; 1 ♀, 1 juv. (ASU), site 2 on S slope, soil sample 2 (0–10 cm deep), 22.08.2016; 2 juv. (ASU), site 2 on S slope, soil sample 4 (0–10 cm deep), 22.08.2016; 1 juv. (ASU), site 2 on S slope, soil sample 5 (litter), 22.08.2016; 1 juv. (ASU), site 1 on N slope, soil sample 1 (0–10 cm deep), 23.08.2016; 1 ♂, 1 juv. (ASU), site 1 on N slope, soil sample 3 (litter), 23.08.2016; 2 ♂♂, 1 ♀, 2 juv. (ASU), site 1 on N slope, soil sample 3 (0–10 cm deep), 23.08.2016; 1 ♂ (ASU), site 1 on N slope, hand sampling, 23.08.2016; 2 ♂♂, 3 ♀♀, 1 juv. (ASU), site 2 on N slope, soil sample 1 (0–10 cm deep), 23.08.2016; 1 juv. (ASU), site 2 on N slope, soil sample 4 (0–10 cm deep), 23.08.2016; 1 juv. (ASU), site 2 on N slope, soil sample 5 (litter), 23.08.2016, all leg. P.N., Kh.N., S.N., V.S.; 1 subadult ♂ (ASU), *Betula pendula* and *Populus tremula* stand on N slope, 51°21'33.8"N, 83°37'23.2"E, 518 m a.s.l., hand sampling, 20.06.2017, leg. P.N.; 1 ♀, 1 subadult ♀, 1 juv. (ASU), site 1 on N slope, hand sampling, 23.06.2017, leg. P.N., Kh.N., A.A., E.A.

######## Distribution.

Central-Palaearctic temperate range: a central Asian species, *L. insolens* has very recently been found in the Omsk Area, Altai Province, and Republic of Altai (Nefediev et al. 2017b, c, d).

######## Remarks.

The above record of *L. insolens*, recently announced at the 17th International Congress of Myriapodology (Nefediev et al. 2017c), can be considered as the first formal record of the species in the Altai Province, SW Siberia. In the study area, the species has significant preference for the southern slope. A single ♂ with aberrant numbers of antennomeres (22+24 vs. 20+20 in original description) was found.

####### Lithobius (Monotarsobius) nordenskioeldii

Taxon classificationAnimaliaLithobiomorphaLithobiidae

Stuxberg, 1876

Lithobius (Monotarsobius) nordenskioeldii – Nefediev et al. 2017c: 13; 2017d: 221, 220: map.

######## Material examined.

1 juv. (ASU), Russia, southwestern Siberia, Altai Province, Charysh District, ca. 4.5 air-km SE of Charyshskoye Village, site 1 on N slope, soil sample 3 (0–10 cm deep), 13.07.2016, leg. Kh.N., S.N., V.S.

######## Distribution and remarks.

Until recently this species was been known only from its *terra typica* in the Krasnoyarsk Province, central Siberia, Russia. New records of *L. nordenskioeldii* in the Altai Province, as announced at the 17th International Congress of Myriapodology (Nefediev et al. 2017c), and in the Republic of Altai (Nefediev et al. 2017d) seems to indicate the low level of species abundance in the Altai region.

####### Lithobius (Monotarsobius)

Taxon classificationAnimaliaLithobiomorphaLithobiidae

sp.

######## Material examined

(all from Russia, southwestern Siberia, Altai Province, Charysh District, ca 4.5 km SE of Charyshskoye Village). 1 juv. (ASU), site 1 on N slope, soil sample 4 (litter), 2.06.2016; 1 ♂ (ASU), site 1 on N slope, soil sample 5 (litter), 2.06.2016; 1 ♂, 2 subadult ♂♂ (ASU), site 1 on N slope, hand sampling, 2.06.2016, all leg. P.N., Kh.N., S.N., V.S.

######## Remarks.

The species identity of this new record is delayed pending an examination of additional material of specimens with similar diagnostic characters from the Republic of Altai.

####### Lithobius vagabundus

Taxon classificationAnimaliaLithobiomorphaLithobiidae

Stuxberg, 1876

Lithobius vagabundus – Nefediev et al. 2017c: 13; 2017d: 219, 218: map.

######## Material examined

(all from Russia, southwestern Siberia, Altai Province, Charysh District, ca. 4.5 km SE of Charyshskoye Village). 1 ♂, 1 subadult ♂ (PSU), foot of S slope of mountain, *Padus avium* and *Populus tremula* stand near brook, hand sampling, 31.05.2016; 1 ♀ (PSU), site 2 on S slope, soil sample 1 (0–10 cm deep), 12.07.2016, leg. Kh.N., S.N., V.S.; 1 ♂, 1 ♀ (PSU), site 1 on N slope, hand sampling, 23.06.2017; 1 ♂ (PSU), site 2 on N slope, hand sampling, 23.06.2017, all leg. P.N., Kh.N., A.A., E.A.

######## Distribution.

Originally described from the Yenisei River basin, Krasnoyarsk Province, central Siberia (Zalesskaja 1978), the species has been found recently in the Altai Province and Republic of Altai (Nefediev et al. 2017c, d), both SW Siberia, Russia.

######## Remarks.

The above finding of *L. vagabundus*, recently announced at the 17th International Congress Myriapodology (Nefediev et al. 2017c), can be considered as the first formal record of the species in southwestern Siberia. In the study region, the species was very rare in all biotopes.

####### Lithobius

Taxon classificationAnimaliaLithobiomorphaLithobiidae

sp.

######## Material examined

(all from Russia, southwestern Siberia, Altai Province, Charysh District, ca. 4.5 km SE of Charyshskoye Village). 1 juv. (ASU), site 1 on N slope, soil sample 2 (litter), 2.06.2016; 1 ♂ (ASU), site 1 on N slope, soil sample 3 (10–20 cm deep), 2.06.2016, all leg. P.N., Kh.N., S.N., V.S.; 1 juv. (ASU), site 1 on S slope, soil sample 3 (0–10 cm deep), 12.07.2016, leg. Kh.N., S.N., V.S.; 2 juv. (ASU), site 1 on N slope, hand sampling, 23.06.2017, all leg. P.N., Kh.N., A.A., E.A.

######## Remarks.

The identification of the above recorded specimens to the species level is impossible due to their early instars or lack of legs.

#### Order Geophilomorpha Pocock, 1895

##### Family Geophilidae Cook, 1895

###### Genus *Arctogeophilus* Attems, 1909

####### Arctogeophilus macrocephalus

Taxon classificationAnimaliaLithobiomorphaLithobiidae

Folkmanová & Dobroruka, 1960

? Arctogeophilus sp. – Byzova and Chadaeva 1965: 337.
Arctogeophilus macrocephalus – Zalesskaja et al. 1982: 189; Nefediev et al. 2017a: 8, 10: map; 2017c: 13; 2017d: 221, 222: map.

######## Material examined

(all from Russia, southwestern Siberia, Altai Province, Charysh District, ca. 4.5 km SE of Charyshskoye Village). 1 juv. (ASU), site 1 on S slope, soil sample 1 (10–20 cm deep), 31.05.2016; 1 juv. (ASU), site 1 on S slope, soil sample 2 (10–20 cm deep), 31.05.2016; 1 juv. (ASU), site 1 on S slope, soil sample 4 (0–10 cm deep), 1.06.2016; 1 juv. (ASU), site 2 on S slope, soil sample 2 (0–10 cm deep), 1.06.2016; 1 juv. (ASU), site 2 on S slope, soil sample 3 (0–10 cm deep), 1.06.2016; 1 juv. (ASU), site 2 on S slope, soil sample 4 (0–10 cm deep), 1.06.2016; 1 juv. (ASU), site 2 on S slope, soil sample 5 (0–10 cm deep), 1.06.2016; 1 ♀ (ASU), site 1 on N slope, hand sampling, 2.06.2016, all leg. P.N., Kh.N., S.N., V.S.; 1 juv. (ASU), site 2 on N slope, soil sample 1 0–10 cm deep), 13.07.2016, leg. Kh.N., S.N., V.S.; 1 ♂, 1 ♀ (ASU), site 1 on S slope, soil sample 1 (0–10 cm deep), 22.08.2016; 1 ♀ (ASU), site 1 on S slope, soil sample 2 (0–10 cm deep), 22.08.2016; 1 juv. (ASU), site 1 on S slope, soil sample 4 (0–10 cm deep), 23.08.2016, all leg. P.N., Kh.N., S.N., V.S.; 2 ♂♂, 1 ♀ (ZMUM), *Betula pendula* and *Populus tremula* stand on N slope, 51°21'33.8"N, 83°37'23.2"E, 518 m a.s.l., hand sampling, 20.06.2017, leg. P.N.

######## Distribution.

Trans-Eurasian temperate range: this species is very widely distributed, ranging from European Russia through Siberia to the Far East of Russian (Zalesskaja et al. 1982). In southwestern Siberia *A. macrocephalus* has been recorded in the Kemerovo and Tomsk areas, Republic of Altai and Altai Province (Byzova and Chadaeva 1965; Zalesskaja et al. 1982; Nefediev et al. 2017a, c, d).

######## Remarks.

Apparently a very euryoecious species, *A. macrocephalus* has currently been recorded mainly from habitats on the southern slope.

##### Family Linotaeniidae Cook, 1904

###### Genus *Strigamia* Gray, 1843

####### Strigamia pusilla

Taxon classificationAnimaliaLithobiomorphaLinotaeniidae

(Sseliwanoff, 1884)

Strigamia pusilla – Nefediev et al. 2017c: 13; 2017d: 223, 222: map.

######## Material examined

(all from Russia, southwestern Siberia, Altai Province, Charysh District, ca. 4.5 km SE of Charyshskoye Village). 1 ♂, 1 juv. (ZMUM), 1 ♂ (ASU), site 1 on N slope, soil sample 5 (0–10 cm deep), 2.06.2016; 1 ♀ (ASU), site 1 on N slope, soil sample 1 (0–10 cm deep), 23.08.2016; 1 juv. (ASU), site 2 on N slope, soil sample 1 (0–10 cm deep), 23.08.2016, all leg. P.N., Kh.N., S.N., V.S.

######## Distribution.

Central-Palearctic temperate range: widespread from Central Europe and the Caucasus, *S. pusilla* is found in the Urals, SW and central Siberia and N Mongolia (Bonato et al. 2012; Poloczek et al. 2016; Nefediev et al. 2017c, d).

######## Remarks.

In the study area, the species was found rarely and on the northern slope only.

####### Strigamia cf. transsilvanica

Taxon classificationAnimaliaLithobiomorphaLinotaeniidae

(Verhoeff, 1928)

Strigamia sp. – Nefediev et al. 2017c: 13.

######## Material examined

(all from Russia, southwestern Siberia, Altai Province, Charysh District, ca. 4.5 km SE of Charyshskoye Village). 1 ♂ (ASU), *Betula pendula* and *Populus tremula* stand, 51°21'33.8"N, 83°37'23.2"E, 518 m a.s.l., hand sampling, 14.07.2015, leg. P.N.; 1 ♂ (ASU), site 2 on S slope, soil sample 5 (0–10 cm deep), 2.06.2016, leg. P.N., Kh.N., S.N., V.S.

######## Distribution.

A central-eastern European species, *S. transsilvanica* appears to be quite widespread in continental Europe from the Alps to the Carpathians and from the Baltic states to mainland Greece. It has been doubtfully reported from Sakhalin (Russia), Japan and Taiwan (Bonato et al. 2012) and recently found in the Rostov-on-Don Area, south of European Russia (Zuev and Evsyukov 2016).

######## Remarks.

Although both specimens resemble *S. transsilvanica*, the study area is far from the known distribution of the species. Aside from the possibility of human introduction of this species in the Charysh District, the presence of a possible undescribed species similar in morphology to *S. transsilvanica* could be tested by molecular methods in the future.

##### Family Schendylidae Cook, 1896

###### Genus *Escaryus* Cook & Collins, 1891

####### Escaryus koreanus

Taxon classificationAnimaliaLithobiomorphaLinotaeniidae

Takakuwa, 1937

Escaryus koreanus – Titova 1972a: 112; 1972b: 135; Pereira and Hoffman 1993: 9; Nefediev et al. 2017a: 11, 12: map; 2017c: 13; 2017d: 222: map.

######## Material examined

(all from Russia, southwestern Siberia, Altai Province, Charysh District, ca. 4.5 km SE of Charyshskoye Village). 1 ♂, 1 ♀ (ZMUM), 5 ♀♀, 5 juv. (ASU), *Betula pendula* and *Populus tremula* stand on N slope, 51°21'33.8"N, 83°37'23.2"E, 518 m a.s.l., 14.07.2015; 1 juv. (ASU), *Lonicera tatarica* on E slope, 51°21'24.9"N, 83°37'24.4"E, 493 m a.s.l., 16.07.2015, all leg P.N.; 1 ♂, 3 ♀♀ (ASU), foot of S slope of mountain, *Padus avium* and *Populus tremula* stand near brook, hand sampling, 31.05.2016; 1 ♀ (ASU), site 1 on S slope, soil sample 3 (10–20 cm deep), 31.05.2016; 2 juv. (ASU), site 1 on N slope, soil sample 3 (litter), 2.06.2016; 2 juv. (ASU), site 1 on N slope, soil sample 3 (10–20 cm deep), 2.06.2016; 1 ♂ (ASU), site 1 on N slope, soil sample 5 (0–10 cm deep), 2.06.2016; 2 ♂♂ (ASU), site 1 on N slope, hand sampling, 2.06.2016; 2 juv. (ASU), site 2 on N slope, soil sample 1 (0–10 cm deep), 2.06.2016; 2 juv. (ASU), site 2 on N slope, soil sample 2 (0–10 cm deep), 2.06.2016; 1 juv. (ASU), site 2 on N slope, soil sample 3 (litter), 2.06.2016; 2 juv. (ASU), site 2 on N slope, soil sample 3 (0–10 cm deep), 2.06.2016; 1 ♂ (ASU), site 2 on N slope, soil sample 5 (litter), 2.06.2016; 1 juv. (ASU), site 2 on N slope, soil sample 5 (0–10 cm deep), 2.06.2016, all leg. P.N., Kh.N., S.N., V.S.; 1 ♂, 1 ♀, 3 juv. (ASU), *Betula pendula* and *Populus tremula* stand on N slope, 51°21'33.8"N, 83°37'23.2"E, 518 m a.s.l., 12.07.2016, leg. P.N.; 1 ♀ (ASU), site 1 on N slope, soil sample 1 (0–10 cm deep), 13.07.2016; 2 ♂♂ (ASU), site 1 on N slope, soil sample 2 (0–10 cm deep), 13.07.2016; 1 ♂ (ASU), site 1 on N slope, hand sampling, 13.07.2016; 1 ♀, 2 juv. (ASU), site 2 on N slope, soil sample 1 (0–10 cm deep), 13.07.2016; 1 ♀ (ASU), site 2 on N slope, soil sample 4 (0–10 cm deep), 13.07.2016; 1 ♀ (ASU), site 2 on N slope, soil sample 5 (0–10 cm deep), 13.07.2016, all leg. Kh.N., S.N., V.S.; 1 juv. (ASU), site 1 on N slope, soil sample 1 (0–10 cm deep), 23.08.2016; 1 ♂ (ASU), site 1 on N slope, soil sample 2 (0–10 cm deep), 23.08.2016; 2 ♀♀, 13 juv. (ASU), site 1 on N slope, soil sample 3 (0–10 cm deep), 23.08.2016; 1 juv., 1 fragm. (ASU), site 2 on N slope, soil sample 1 (0–10 cm deep), 23.08.2016; 1 ♀ (ASU), site 2 on N slope, soil sample 2 (0–10 cm deep), 23.08.2016; 1 juv., 1 fragm. (ASU), site 2 on N slope, soil sample 4 (0–10 cm deep), 23.08.2016; 1 ♂ (ASU), site 2 on N slope, soil sample 5 (0–10 cm deep), 23.08.2016; 2 ♂♂, 2 ♀♀, 1 juv. (ASU), site 2 on N slope, hand sampling, 23.08.2016, all leg. P.N., Kh.N., S.N., V.S.; 1 subadult ♂, 4 ♀♀, 1 juv. (ASU), *Betula pendula* and *Populus tremula* stand on N slope, 51°21'33.8"N, 83°37'23.2"E, 518 m a.s.l., hand sampling, 20.06.2017, leg. P.N.

######## Distribution.

Trans-Palaearctic: originally described from Korea, the species is widespread throughout Asian Russia; also known from Armenia, Azerbaijan, Kazakhstan, Tadzhikistan, Turkmenistan and Uzbekistan (Bonato et al 2016); in SW Siberia *E. koreanus* was formally recorded in the Kemerovo and Tomsk areas, Altai Province and Republic of Altai (Titova 1972a, b; Nefediev et al. 2017a, c, d).

######## Remarks.

In the study region, *E. koreanus* appears to be found mainly on the northern slope.

####### Escaryus retusidens

Taxon classificationAnimaliaLithobiomorphaLinotaeniidae

Attems, 1904

Escaryus retusidens – Titova 1972a: 110; 1972b: 135; Pereira and Hoffman 1993: 9; Volkova 2016: 675; Nefediev et al. 2017a: 11, 13: map; 2017c: 13; 2017d: 222: map.

######## Material examined

(all from Russia, southwestern Siberia, Altai Province, Charysh District, ca. 4.5 km SE of Charyshskoye Village). 1 ♂, 1 ♀ (ZMUM), 2 ♂♂, 4 juv. (ASU), *Betula pendula* and *Populus tremula* stand on N slope, 51°21'33.8"N, 83°37'23.2"E, 518 m a.s.l., 14.07.2015, leg P.N.; 1 ♂ (ASU), foot of S slope of mountain, *Padus avium* and *Populus tremula* stand near brook, hand sampling, 31.05.2016; 2 ♀♀, 3 juv. (ASU), site 1 on S slope, hand sampling, 31.05.2016; 2 juv. (ASU), site 1 on S slope, soil sample 1 (10–20 cm deep), 31.05.2016; 1 ♀ (ASU), site 1 on S slope, soil sample 1 (20–30 cm deep), 31.05.2016; 3 ♀♀ (ASU), site 1 on S slope, soil sample 3 (10–20 cm deep), 31.05.2016; 1 ♀, 3 juv. (ASU), site 1 on S slope, soil sample 3 (20–30 cm deep), 31.05.2016; 1 juv. (ASU), site 1 on S slope, soil sample 4 (20–30 cm deep), 1.06.2016; 1 juv. (ASU), site 1 on S slope, soil sample 5 (0–10 cm deep), 1.06.2016; 3 juv. (ASU), site 1 on S slope, soil sample 5 (10–20 cm deep), 1.06.2016; 1 ♂, 1 juv. (ASU), site 1 on S slope, soil sample 5 (20–30 cm deep), 1.06.2016; 1 ♂ (ASU), S slope between site 1 and site 2, broad gully with *Padus avium*, hand sampling, 1.06.2016; 1 juv. (ASU), site 2 on S slope, soil sample 1 (0–10 cm deep), 1.06.2016; 1 fragm. (ASU), site 2 on S slope, soil sample 1 (10–20 cm deep), 1.06.2016; 2 juv. (ASU), site 2 on S slope, soil sample 2 (0–10 cm deep), 1.06.2016; 1 juv. (ASU), site 2 on S slope, soil sample 2 (10–20 cm deep), 1.06.2016; 1 juv. (ASU), site 2 on S slope, soil sample 3 (0–10 cm deep), 1.06.2016; 1 juv. (ASU), site 2 on S slope, soil sample 5 (0–10 cm deep), 1.06.2016; 1 juv. (ASU), site 2 on S slope, hand sampling, 1.06.2016; 2 juv. (ASU), site 1 on N slope, soil sample 1 (0–10 cm deep), 2.06.2016; 2 ♀♀, 1 juv. (ASU), site 1 on N slope, soil sample 2 (0–10 cm deep), 2.06.2016; 2 juv. (ASU), site 1 on N slope, soil sample 2 (10–20 cm deep), 2.06.2016; 2 ♂♂, 2 ♀♀, 2 juv. (ASU), site 1 on N slope, soil sample 3 (0–10 cm deep), 2.06.2016; 1 ♂, 1 juv. (ASU), site 1 on N slope, soil sample 3 (10–20 cm deep), 2.06.2016; 1 ♀, 2 juv. (ASU), site 1 on N slope, soil sample 4 (0–10 cm deep), 2.06.2016; 1 ♂ (ASU), site 1 on N slope, soil sample 4 (10–20 cm deep), 2.06.2016; 1 ♂, 3 ♀♀ (ASU), site 1 on N slope, soil sample 5 (0–10 cm deep), 2.06.2016; 1 ♀ (ASU), site 1 on N slope, hand sampling, 2.06.2016; 1 ♀, 1 juv. (ASU), site 2 on N slope, soil sample 1 (0–10 cm deep), 2.06.2016; 3 juv. (ASU), site 2 on N slope, soil sample 2 (0–10 cm deep), 2.06.2016; 2 ♂♂, 2 ♀♀, 1 juv. (ASU), site 2 on N slope, soil sample 3 (0–10 cm deep), 2.06.2016; 1 ♀, 3 juv. (ASU), site 2 on N slope, soil sample 4 (0–10 cm deep), 2.06.2016; 1 juv. (ASU), site 2 on N slope, soil sample 4 (10–20 cm deep), 2.06.2016, all leg. P.N., Kh.N., S.N., V.S.; 1 ♀ (ASU), site 1 on N slope, hand sampling, 22.06.2016, leg. Kh.N.; 1 adult specimen (ASU), *Betula pendula* and *Populus tremula* stand on N slope, 51°21'33.8"N, 83°37'23.2"E, 518 m a.s.l., 12.07.2016, leg. P.N.; 1 juv. (ASU), site 1 on S slope, soil sample 1 (0–10 cm deep), 12.07.2016; 2 juv. (ASU), site 1 on S slope, soil sample 5 (10–20 cm deep), 12.07.2016; 1 ♂, 2 juv. (ASU), site 1 on N slope, soil sample 1 (10–20 cm deep), 13.07.2016; 1 fragm. (ASU), site 1 on N slope, soil sample 3 (0–10 cm deep), 13.07.2016; 2 ♀♀ (ASU), site 2 on N slope, soil sample 1 (0–10 cm deep), 13.07.2016; 1 juv. (ASU), site 2 on N slope, soil sample 2 (0–10 cm deep), 13.07.2016; 1 ♀, 1 juv. (ASU), site 2 on N slope, soil sample 4 (0–10 cm deep), 13.07.2016; 2 ♀♀ (ASU), site 2 on N slope, soil sample 4 (10–20 cm deep), 13.07.2016, all leg. Kh.N., S.N., V.S.; 1 juv., 2 fragm. (ASU), site 1 on S slope, soil sample 1 (10–20 cm deep), 22.08.2016; 1 fragm. (ASU), site 1 on S slope, soil sample 2 (10–20 cm deep), 22.08.2016; 1 ♀, 2 juv., 1 fragm. (ASU), site 1 on S slope, soil sample 4 (0–10 cm deep), 23.08.2016; 1 ♂ (ASU), site 2 on S slope, soil sample 5 (0–10 cm deep), 22.08.2016; 1 ♂, 2 ♀♀, 2 juv. (ASU), site 1 on N slope, soil sample 2 (0–10 cm deep), 23.08.2016; 1 juv. (ASU), site 1 on N slope, soil sample 4 (0–10 cm deep), 23.08.2016; 2 ♀♀, 1 juv. (ASU), site 1 on N slope, soil sample 5 (0–10 cm deep), 23.08.2016; 3 ♂♂, 1 ♀, 1 juv. (ASU), site 2 on N slope, soil sample 1 (0–10 cm deep), 23.08.2016; 1 ♀ (ASU), site 2 on N slope, soil sample 2 (0–10 cm deep), 23.08.2016; 2 ♀♀, 1 juv. (ASU), site 2 on N slope, soil sample 3 (0–10 cm deep), 23.08.2016; 1 ♀, 1 juv., 2 fragm. (ASU), site 2 on N slope, soil sample 3 (10–20 cm deep), 23.08.2016; 2 ♂♂, 1 ♀, 1 juv. (ASU), site 2 on N slope, soil sample 5 (0–10 cm deep), 23.08.2016; 1 ♀ (ASU), site 2 on N slope, hand sampling, 23.08.2016, all leg. P.N., Kh.N., S.N., V.S.; 3 ♂♂, 4 ♀♀, 3 juv. (ASU), *Betula pendula* and *Populus tremula* stand on N slope, 51°21'33.8"N, 83°37'23.2"E, 518 m a.s.l., 20.06.2017, leg. P.N.; 1 ♂ (ASU), site 1 on N slope, hand sampling, 23.06.2017, leg. P.N., Kh.N., A.A., E.A.

######## Distribution.

Central-Eastern-Palaearctic subboreal range: originally described from Kyrgyzstan, the species is widely distributed in Eurasia, spanning from the Black Sea region in the west through eastern Kazakhstan to Cisamuria in the east (Titova 1972b). In Siberia *E. retusidens* has been known from the Kemerovo Area, Altai Province, and Republic of Altai (Nefediev et al. 2017a, c, d).

######## Remarks.

In the study area, *E. retusidens* inhabits both slopes, and is one of the most dominant species.

## Results and discussion

The myriapod fauna of the study area comprises at least 19 species from 10 genera, 8 families, 5 orders and two classes (Diplopoda and Chilopoda).

The species richness in the millipede assemblages was found to be very low and similar on both slopes (I_J_ = 0.83). Thus, 5 diplopod species are known to occur on both slopes (*Megaphyllum sjaelandicum*, *Sibiriulus latisupremus*, *Orinisobates sibiricus*, *Schizoturanius clavatipes* and *Altajosoma* sp.), whereas *Leptoiulus tigirek* inhabits the northern slope only (Table 1).

**Table 1.** Species composition and species richness in Chilopoda and Diplopoda assemblages in the study area.

Species	S slope		N slope	
	site 1	site 2	site 1	site 2
*Megaphyllum sjaelandicum* (Meinert, 1868)	+	+	–	+
*Sibiriulus latisupremus* Mikhaljova, Nefediev & Nefedieva, 2014	+	+	+	+
*Orinisobates sibiricus* (Gulička, 1963)	+	+	+	+
*Leptoiulus tigirek* Mikhaljova, Nefediev, Nefedieva & Dyachkov, 2015	–	–	+	+
*Schizoturanius clavatipes* (Stuxberg, 1876)	+	+	+	+
*Altajosoma* sp.	+	+	+	+
Lithobius (Ezembius) ostiacorum Stuxberg, 1876	+	–	+	+
Lithobius (Ezembius) proximus Sseliwanoff, 1880	–	+	+	+
Lithobius (Ezembius) sibiricus Gerstfeldt, 1858	+	+	+	+
Lithobius (Monotarsobius) curtipes C.L. Koch, 1847	+	+	+	+
Lithobius (Monotarsobius) insolens Dányi & Tuf, 2012	+	+	+	+
Lithobius (Monotarsobius) nordenskioeldii Stuxberg, 1876	–	–	+	–
Lithobius (Monotarsobius) sp.	–	–	+	–
*Lithobius vagabundus* Stuxberg, 1876	–	+	+	+
*Arctogeophilus macrocephalus* Folkmanová & Dobroruka, 1960	+	+	+	+
*Strigamia pusilla* (Sseliwanoff, 1884)	–	–	+	+
Strigamia cf. transsilvanica (Verhoeff, 1928)	–	+	–	–
*Escaryus koreanus* Takakuwa, 1937	+	–	+	+
*Escaryus retusidens* Attems, 1904	+	+	+	+
Species richness in each site	12	13	17	16
Species richness on each slope	15		17	
Total species richness on both slopes	19			

The total species richness in the centipede assemblages is twice as high compared to the millipede one, with 10 and 12 species recorded on the southern and northern slopes, respectively. Most Chilopoda species are common to both slopes, namely, Lithobius (Ezembius) ostiacorum, L. (E.) proximus, L. (E.) sibiricus, L. (Monotarsobius) curtipes, L. (M.) insolens, *L. vagabundus*, *Arctogeophilus macrocephalus*, *Escaryus koreanus* and *E. retusidens*. However, the similarity in species composition between the study slopes is weak (I_J_ = 0.69). Thus, a single species was recorded only on the southern slope (Strigamia cf. transsilvanica) while three species dwell only on the northern slope (L. (M.) nordenskioeldii, L. (M.) sp. and *Strigamia pusilla*) (Table 1).

The julid *L. tigirek*, which has recently been included in the Red Data Book of the Altai Province (Nefediev 2016), has been collected outside its *terra typica* for the first time, thus also expanding the eastern range limit of the species (Figure 6). The julid *S. latisupremus* has previously been known from the Smolenskoe and Altaiskoe districts in the Altai Province and from the Shebalino District in the Republic of Altai (Mikhaljova et al. 2014). The current record of the species is the westernmost known to date (Figure 7). The species identity of *Altajosoma* sp. is delayed pending a revision of the variation in *Altajosoma bakurovi bakurovi* (Shear, 1990), which the currently recorded diplomaragnid is close to in the shape of colpocoxites of posterior gonopods and in their distal parts, but differs significantly in the large posterior angiocoxal processes.

Five lithobiids, L. (E.) proximus, L. (M.) insolens, L. (E.) ostiacorum, *L. vagabundus* and L. (M.) nordenskioeldii, are new to the Altai Province, while the three latter are also recorded in southwestern Siberia for the first time; the linotaeniid Strigamia cf. transsilvanica is reported from Asian Russia for the first time too.

**Figure 8.**
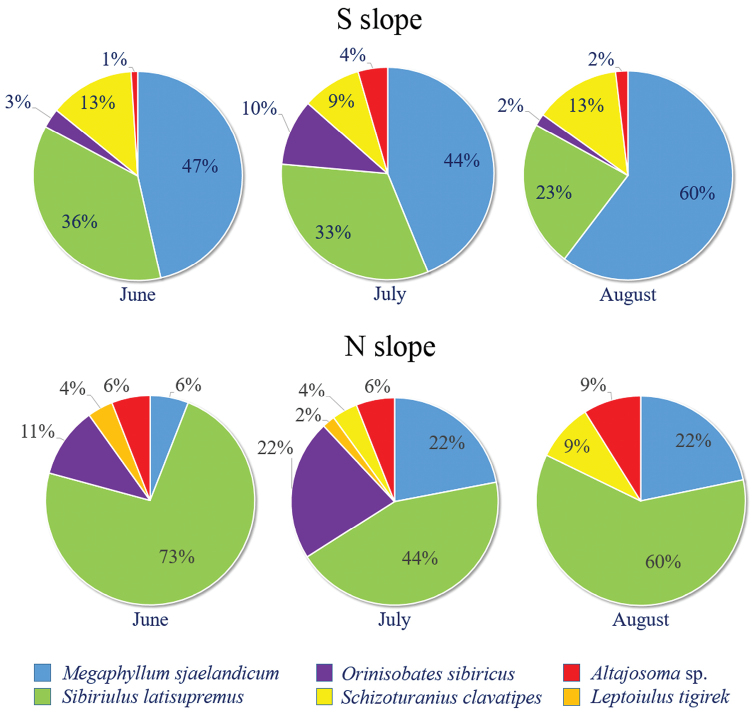
The species diversity of millipedes on the southern and northern slopes.

**Figure 9.**
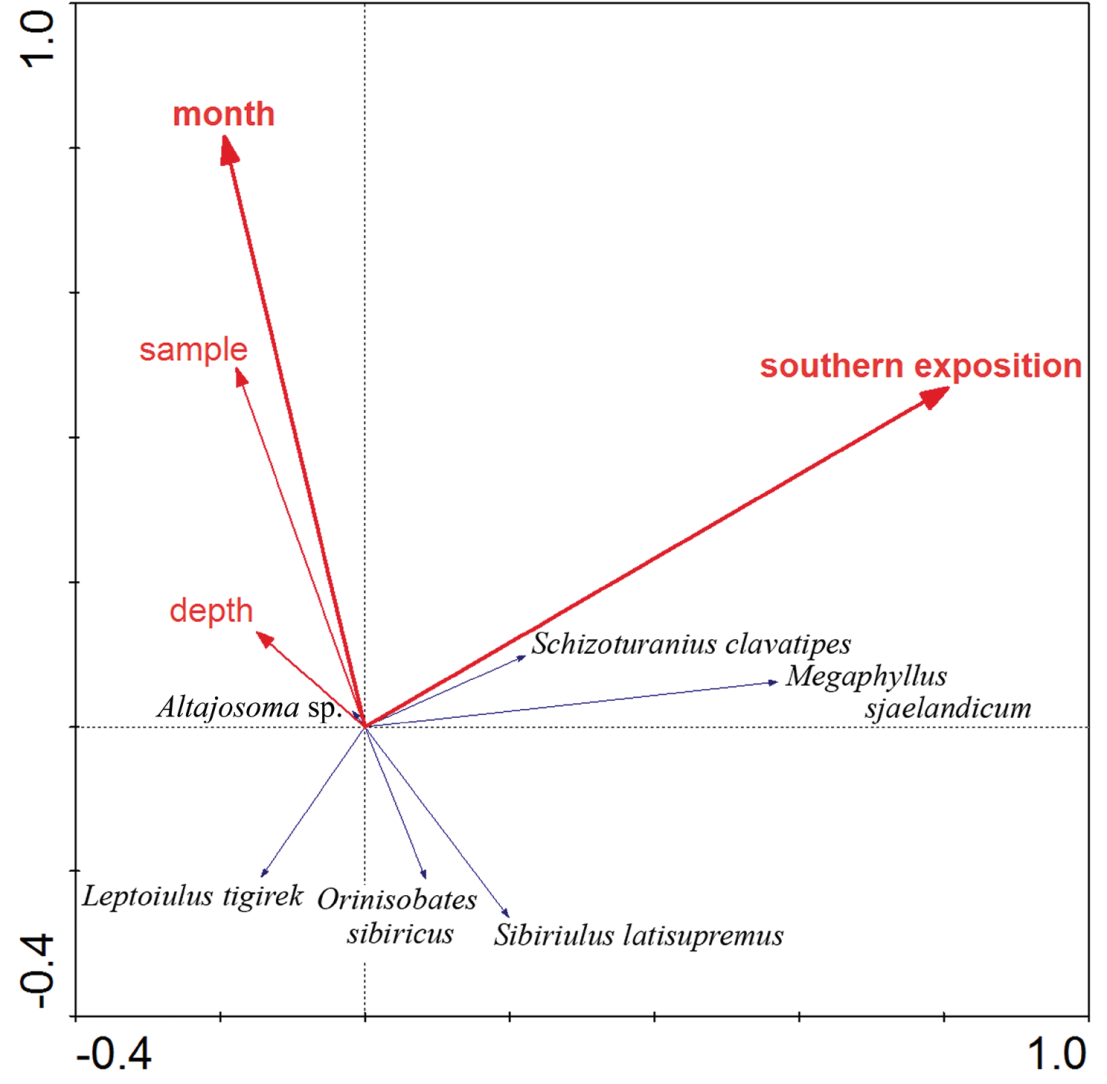
RDA ordination biplot of the distribution patterns of millipedes in soil samples on the study slopes. Environmental variables significantly contributing to the prediction are in bold. The whole model is statistically significant (F = 4.73, p = 0.002) and explains 20.3 % of variability of species data, the X-axis explains 16.5 %.

The species diversity of Diplopoda is very low on both slopes. The julid *M. sjaelandicum* predominates on the dry southern slope, ranging from 44 to 60 % of the total millipede abundance, whereas *S. latisupremus* tends to dominate on the more humid northern slope, ranging from 44 to 73 % of the total diplopod abundance (Figure 8). The latter species may also be considered as a codominant species on the southern slope (23–36 % of the total millipede abundance), while the rest of the millipede species are rare or very rare on the southern slope. Codominants of the northern slope appear to be *M. sjaelandicum* and *O. sibiricus* with 22 % of the diplopod abundance. The RDA model also reveals the pattern of millipede distribution (Figure 9) explaining 20.3 % of the variability in species data. Of the tested environmental variables, slope exposure (south/north) and time of sampling (month) are significant (F = 9.88, p = 0.002 and F = 3.42, p = 0.018, respectively). Of the recorded species, *M. sjaelandicum* and *S. clavatipes* predominate on the southern slope.

**Figure 10.**
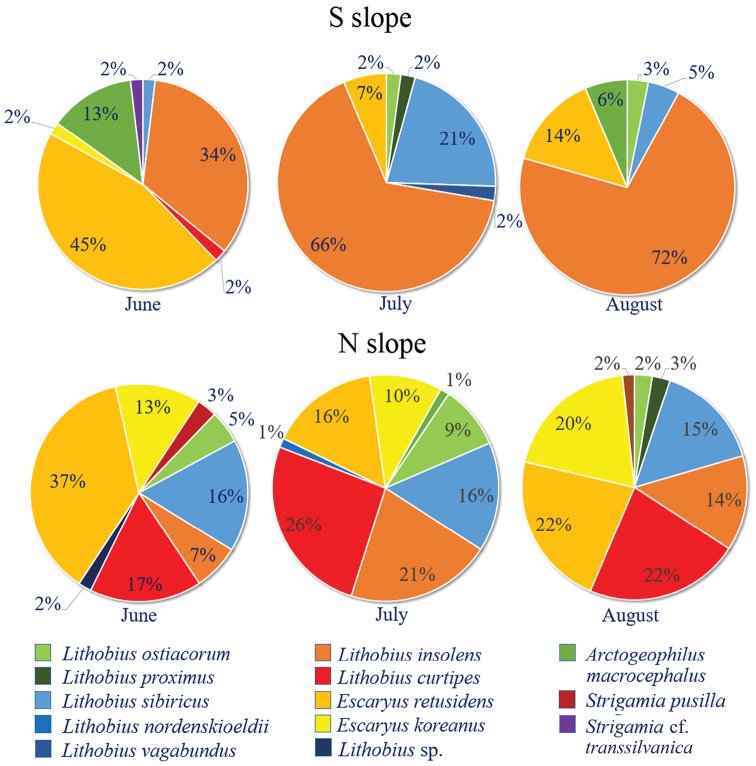
The species diversity of centipedes on the southern and northern slopes.

**Figure 11.**
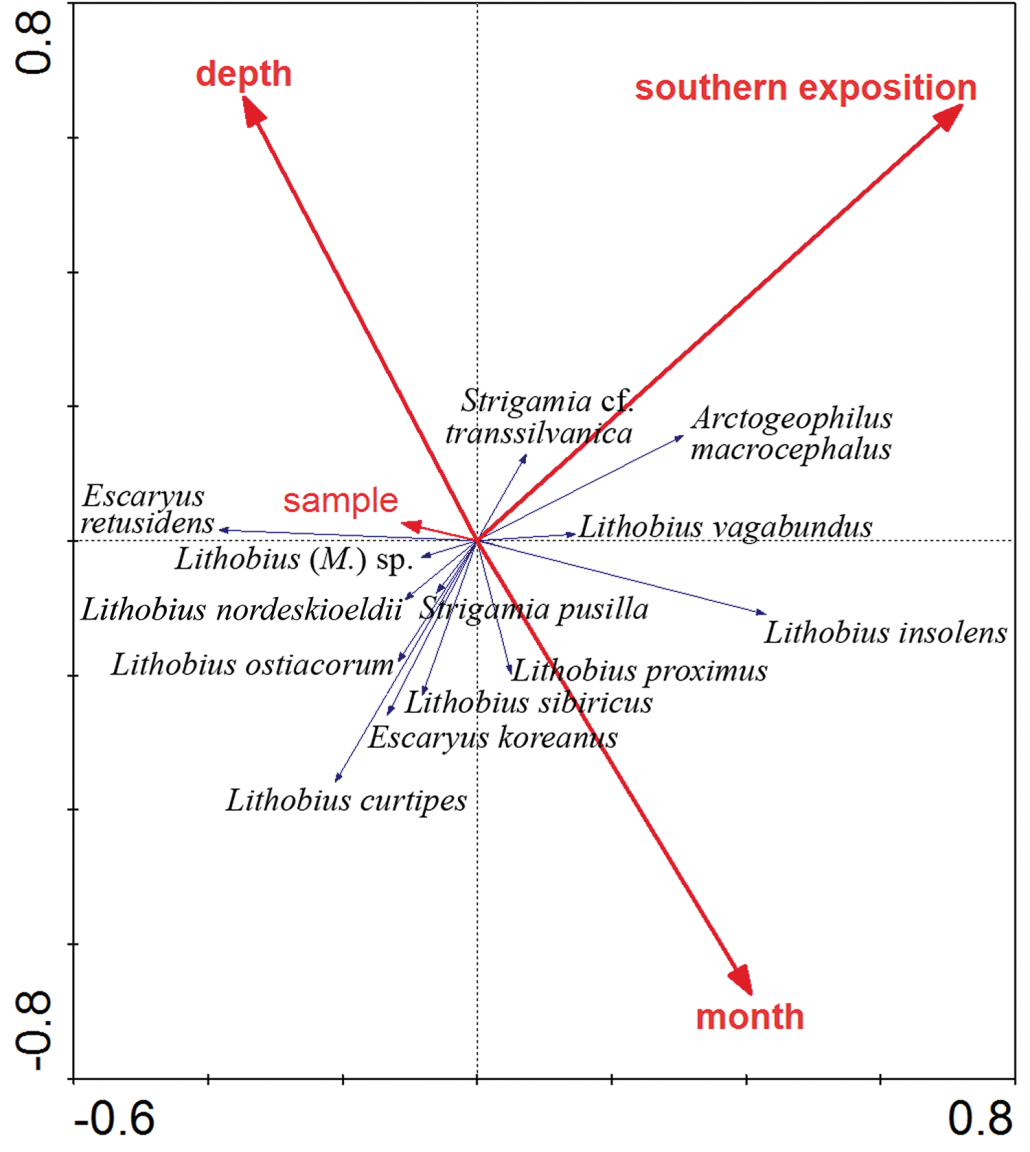
RDA ordination biplot of the distribution patterns of centipedes in soil samples on the study slopes. Environmental variables significantly contributing to the prediction are in bold. The whole model is statistically significant (F = 4.12, p = 0.002) and explains 15.2 % of variability of species data, the X-axis explains 10.3 %.

Species diversity of Chilopoda is low on the southern slope: two species predominate, in particular, L. (M.) insolens, ranging from 34 to 72 % of the total chilopod abundance, and *E. retusidens* with 45 % of the total centipede abundance in June, likewise L. (E.) sibiricus codominating there (21 % in July); the rest of the centipede species are rare or very rare on the southern slope (Figure 10). On the northern slope, the centipede community is more similar to that on the southern slope: five dominant or codominant species – *E. retusidens*, *E. koreanus*, *L. (E.)* *sibiricus*, L. (M.) curtipes and L. (M.) insolens – inhabit the northern slope. The RDA model confirms this pattern of centipede distribution (Figure 11) explaining 15.2 % of variability in its distribution. Of the tested environmental variables, slope exposure (south/north), depth of soil sample and time of sampling (month) are significant (F = 7.28, p = 0.002; F = 5.54, p = 0.002; and F = 2.55, p = 0.032, respectively). Of the recorded species, *A. macrocephalus* and L. (M.) insolens predominate on the southern slope, whereas several of the above mentioned species predominate on the northern one.

**Figure 12.**
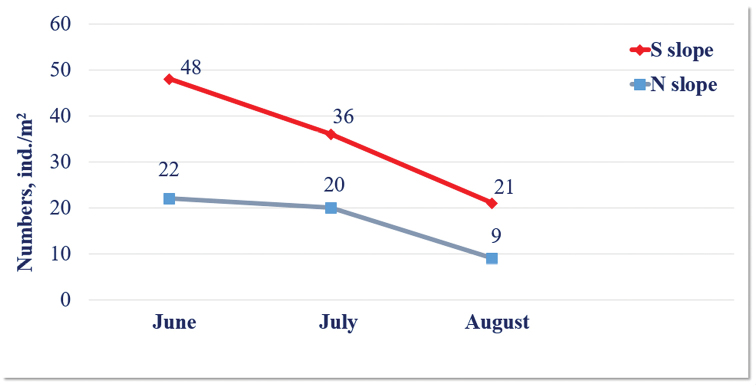
The seasonal dynamics of Diplopoda density on study slopes.

**Figure 13.**
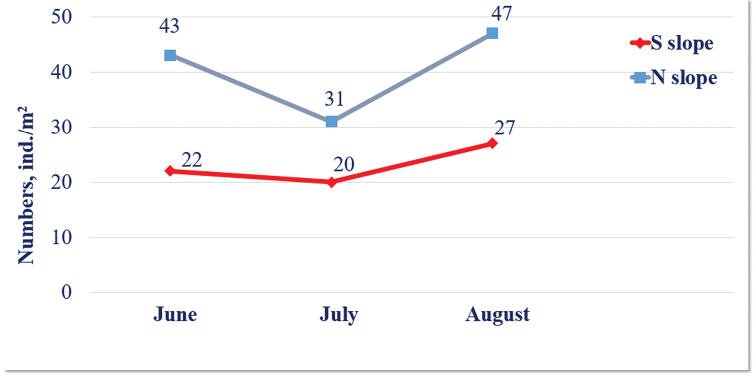
The seasonal dynamics of Chilopoda density on study slopes.

The density of millipedes on the southern slope is twice as high compared to the northern slope. The seasonal dynamics of diplopod numbers range from 21 ± 4.4 to 48 ± 10.8 ind./m² on the southern slope, and from 9 ± 1.2 to 22 ± 13.6 ind./m² on the northern one, gradually declining from June to August in both habitat types (Figure 12). Of the recorded species, abundance of the only julid, *S. latisupremus*, are significantly affected by the time of sampling as the population decreases from June to August (GLM: F = 6.92, p = 0.010). The numbers of centipedes on the northern slope are twice as high compared to the southern one. The seasonal dynamics of Chilopoda density ranges from 20 ± 6.8 to 27 ± 19.6 ind./m² on the southern slope, and from 31 ± 0.0 to 47 ± 11.6 ind./m² on the northern one, the highest being in June and August and the lowest in July in both habitat types (Figure 13).

The age structure will be considered here, using the dominant species as an example. Thus, in the age structure of the julid *M. sjaelandicum* population on the southern slope, juveniles predominated during the summer, and their abundance varied from 100 % of the population in June to 70 % in July and August. In contrast, in the julid *S. latisupremus*, overwintering adults predominated at the beginning of summer (with 75 % of the population), producing juveniles, which started to prevail in the middle of summer (with 76 % of the population).

The age structure in the population of the lithobiid L. (M.) insolens is as follows: adults predominate at the beginning of summer on both slopes, ranging from 70 to 100 % of the population, while young individuals emerge in the middle of summer in amounts equal to the total numbers of males and females, and this ratio is maintained until the late summer. The sex ratio is close to 50:50 during summer on both slopes, but on the southern slope only females exceed males twice over by the end of summer. In the -age structure of *E. retusidens* on the southern slope, the abundance of juveniles is 3 times higher than in adults. On the northern slope, the ratio of adults and juveniles is equal at the beginning of summer, while in the middle and late summer adults start to prevail to become twice as abundant. For adults, the females steady prevailed, outnumbering males from 2 to 5 times throughout the season in both habitats.

**Figure 14.**
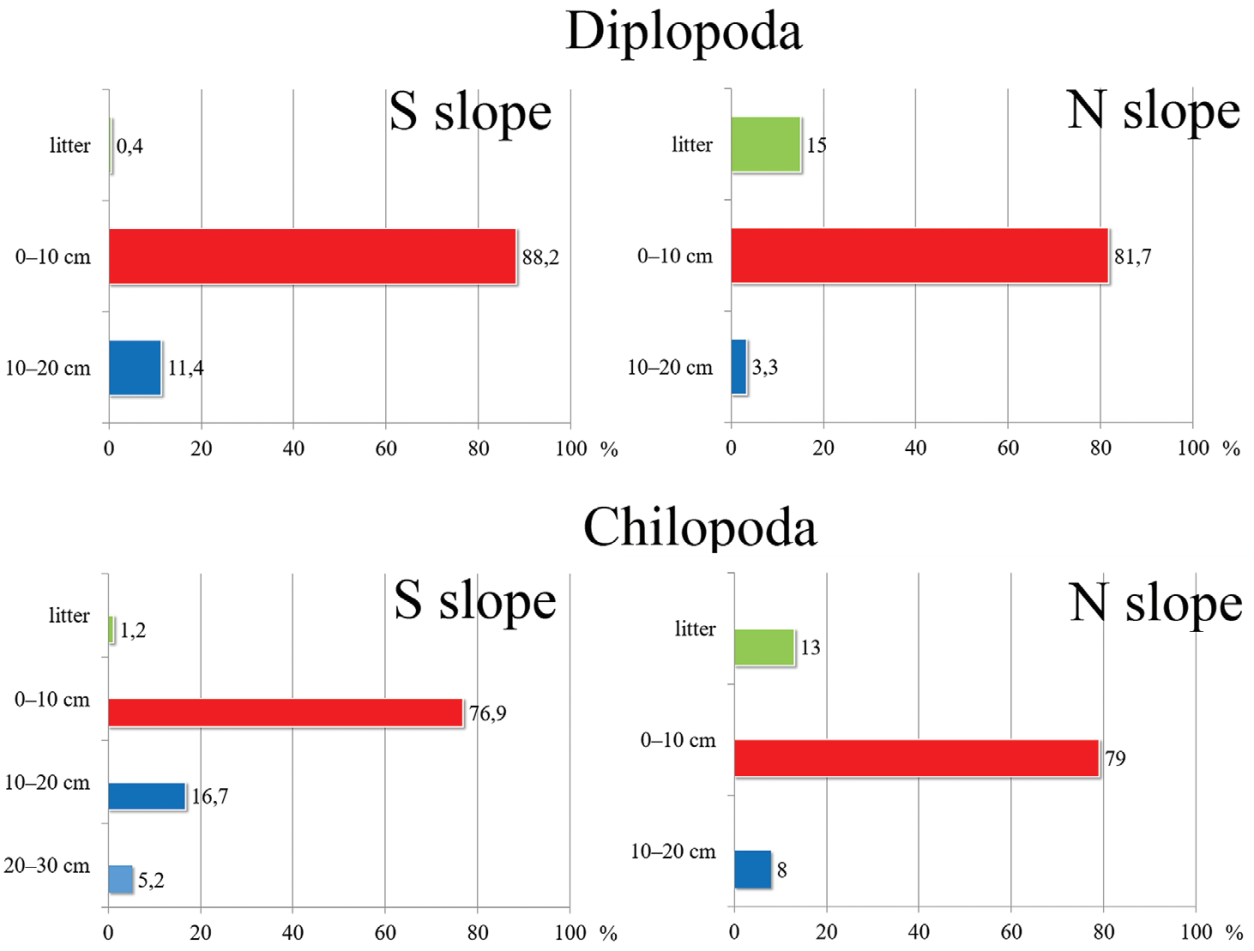
The distribution of myriapods along soil profile on both slopes.

Regarding the vertical distribution in the soil profile, more than 80 % of millipedes prefer the upper soil layer to a depth of 10 cm on both slopes. Diplopods are very rare in the litter, especially on the dry southern slope (where they numbered less than 1 %), but the numbers are about 15 % more on the humid northern slope, with maximum penetration in depth to no more than 20 cm (Figure 14). With regard to the vertical distribution in the soil profile in centipedes, we observe the preference of chilopods to the upper soil layer. Thus, approximately 80 % of centipedes of the total chilopod abundance has been reported from the top 10 cm layer on both study slopes, with the maximum penetration in depth to no more than 30 cm. Centipedes are very rare in the litter, accounting for about 1 % on the dry southern slope and about 13 % on the more humid northern one (Figure 14). As the depth of the sample is a significant variable for RDA model, we tested its power to predict the distribution of individual species. Abundances of the geophilomorph *E. retusidens* and the lithobiomorph L. (M.) curtipes are the only species significantly affected by depth of sample. The geophilomorph prefers deeper soil layers and the lithobiomorph prioritizes the surface and upper soil layers (GLM: F = 6.41, p = 0.013 and F = 4.01, p = 0.048, respectively). This is not surprising, as the preference for the upper layers of soil by L. (M.) curtipes is well known (Tuf 2002, 2015). The ability of geophilomorphs to penetrate to deeper soil layers is documented and also recorded, using subterranean pitfall traps, too (Tuf et al. 2017).

## Conclusions

1. The species richness of millipedes is found to be very low in both habitat types studied, on the northern and southern slopes, whereas the centipede species richness is assessed as twice as high. The total richness comprises at least 19 species, belonging to ten genera, eight families, five orders, and two classes.

2. The new faunistic records for two millipede species, *Megaphyllum sjaelandicum* and *Sibiriulus latisupremus*, clarify their distribution areas. Two lithobiid species, Lithobius (Ezembius) proximus and L. (Monotarsobius) insolens, are new to the Altai Province, while L. (E.) ostiacorum, *L. vagabundus* and L. (M.) nordenskioeldii are recorded here in southwestern Siberia for the first time. A species of *Strigamia* which is morphologically similar to *S. transsilvanica* was found in the study area. Two species from two genera, *Altajosoma* and *Lithobius*, are likely to be new to science, but their descriptions are delayed pending further information.

3. Two species predominate on the southern slope (*M. sjaelandicum* and L. (M.) insolens), and six species are dominant or codominant on the northern one (*S. latisupremus*, *Escaryus retusidens*, *E. koreanus*, L. (E.) sibiricus, L. (M.) curtipes and L. (M.) insolens). Thus, species diversity of millipedes is very low on both slopes, while in centipedes it is low only on the southern slope.

4. The density of millipedes on the southern slope is twice as high compared to the northern one, gradually declining from June to August in both habitat types. In contrast in centipedes, the numbers on the northern slope are twice as high compared to the southern one, with the minimum in mid-summer on both slopes.

5. The age structure of the dominant species is as follows: in *M. sjaelandicum*, juveniles predominated during summer; in *S. latisupremus*, overwintered adults predominate at the beginning of summer (with 75 % of total species abundance), juveniles start to prevail in the middle of summer (with 76 % of total species abundance); in L. (M.) insolens the sex ratio is 50:50; adults predominate in June, while juveniles emerge in the middle of summer in amounts equal to adults; in *E. retusidens* females outnumber males 2–5 times during the whole season in both habitat types.

6. The distribution of myriapods in the soil profile shows that millipedes and centipedes prefer the upper soil layer to 10 cm deep (about 80 % of total myriapod abundance) with the litter more populated on the northern slope, containing from 13 to 15 % of the fauna, and the maximum penetration in depth to no more than 20 cm in millipedes and 30 cm in centipedes. The only geophilomorph centipede, *E. retusidens*, prefers deeper soil layers.

## Supplementary Material

XML Treatment for Leptoiulus tigirek

XML Treatment for Megaphyllum sjaelandicum

XML Treatment for Sibiriulus latisupremus

XML Treatment for Orinisobates sibiricus

XML Treatment for Altajosoma

XML Treatment for Schizoturanius clavatipes

XML Treatment for Lithobius (Ezembius) ostiacorum

XML Treatment for Lithobius (Ezembius) proximus

XML Treatment for Lithobius (Ezembius) sibiricus

XML Treatment for Lithobius (Monotarsobius) curtipes

XML Treatment for Lithobius (Monotarsobius) insolens

XML Treatment for Lithobius (Monotarsobius) nordenskioeldii

XML Treatment for Lithobius (Monotarsobius)

XML Treatment for Lithobius vagabundus

XML Treatment for Lithobius

XML Treatment for Arctogeophilus macrocephalus

XML Treatment for Strigamia pusilla

XML Treatment for Strigamia cf. transsilvanica

XML Treatment for Escaryus koreanus

XML Treatment for Escaryus retusidens
